# Supplementary material for: Physical neural networks using sharpness-aware training
Source: Nat Commun. 2026 Jan 19;17:1766. doi: 10.1038/s41467-026-68470-9 (PMC12917175; doi:10.1038/s41467-026-68470-9)
Supplement: Supplementary file 1 — Supplementary Information [file 41467_2026_68470_MOESM1_ESM.pdf]

Supplementary Notes for 'Physical Neural Networks using Sharpness-Aware Training'

1.Theorem of sharpness-aware training 2

1.1. Theorem 2

1.2. Hyperparameter α sensitivity analysis 3

2.Evaluate the trained model's robustness 4

3.Plot the neural network 3D loss landscape 5

4.MRR-based PNN experiment details 5

4.1. Simulation validation. 5

4.2. Single MRR experimental validation. 10

4.3. Large scale MRR experimental validation. 11

4.4. Extend the verification to CIFAR-10 dataset. 15

5.MZI-based PNNs simulation details 18

6.Diffractive optics-based NNs experiment details 22

6.1. Experimental setup details and basic characterization 22

6.2. Details about training the neural network 25

6.3. Joint optimization 27

6.4. Full neural network inference capability simulation 28

References 30

1.Theorem of sharpness-aware training

1.1. Theorem

In the main text, we propose to modify the loss function from $L(\mathbf{y}, \mathbf{y}_{\text{target}}; \mathbf{\Theta})$ to,

$$L_1 = L(\mathbf{y}, \mathbf{y}_{\text{target}}; \mathbf{\Theta}) + \alpha \left\| \frac{\partial L(\mathbf{y}, \mathbf{y}_{\text{target}}; \mathbf{\Theta})}{\partial \mathbf{\Theta}} \right\|_2 \tag{1}$$

The first term, $L(\mathbf{y}, \mathbf{y}_{\text{target}}; \mathbf{\Theta})$ in the loss function seeks to find the loss minima, while the second term, $\left\| \frac{\partial L(\mathbf{y}, \mathbf{y}_{\text{target}}; \mathbf{\Theta})}{\partial \mathbf{\Theta}} \right\|_2$, which reflects the sensitivity of the objective function to changes in the parameters, aims to identify parameters that lie in neighborhoods with uniformly low loss values. The hyperparameter α indicates how large the penalty of the regularization should be added to the initial loss function.

However, directly calculating the second term $\left\| \frac{\partial L(\mathbf{y}, \mathbf{y}_{\text{target}}; \mathbf{\Theta})}{\partial \mathbf{\Theta}} \right\|_2$ gradient will inevitably calculate the Hessian matrix

$\mathcal{H}(\cdot) = \frac{\partial^2 L}{\partial \mathbf{\Theta}^2}$,

$$\frac{\partial \left\| \frac{\partial L(\mathbf{y}, \mathbf{y}_{\text{target}}; \mathbf{\Theta})}{\partial \mathbf{\Theta}} \right\|_2}{\partial \mathbf{\Theta}} = \frac{\partial^2 L(\mathbf{\Theta})}{\partial \mathbf{\Theta}^2} \cdot \frac{\partial L / \partial \mathbf{\Theta}}{\| \partial L / \partial \mathbf{\Theta} \|_2} \tag{2}$$

Therefore, the computational complexity increases from $O(n)$ to $O(n^2)$ due to the calculation of Hessian matrix. Here, n denotes the neural network parameter number.

This problem can be solved by approximating the Hessian matrix through first-order Taylor expansion [1-2],

$$L(\mathbf{\Theta} + \Delta\mathbf{\Theta}) \approx L(\mathbf{\Theta}) + \frac{\partial L(\mathbf{\Theta})}{\partial \mathbf{\Theta}} \cdot \Delta\mathbf{\Theta} \tag{3}$$

Take the derivative over $\mathbf{\Theta}$ on both sides,

$$\frac{\partial L(\mathbf{\Theta} + \Delta\mathbf{\Theta})}{\partial \mathbf{\Theta}} \approx \frac{\partial L(\mathbf{\Theta})}{\partial \mathbf{\Theta}} + \frac{\partial^2 L(\mathbf{\Theta})}{\partial \mathbf{\Theta}^2} \cdot \Delta\mathbf{\Theta} \tag{4}$$

Therefore, we can assign $\Delta\mathbf{\Theta} = r \frac{\partial L / \partial \mathbf{\Theta}}{\| \partial L / \partial \mathbf{\Theta} \|_2}$, and Equation (2) can be simplified into,

$$\frac{\partial \left\| \frac{\partial L(\mathbf{y}, \mathbf{y}_{\text{target}}; \mathbf{\Theta})}{\partial \mathbf{\Theta}} \right\|_2}{\partial \mathbf{\Theta}} \approx \frac{1}{r} \left(\frac{\partial L(\mathbf{\Theta} + \Delta\mathbf{\Theta})}{\partial \mathbf{\Theta}} - \frac{\partial L(\mathbf{\Theta})}{\partial \mathbf{\Theta}} \right) \tag{5}$$

Overall, the differentiation of loss function L_1 can be expressed as,

$$\frac{\partial L_1(\mathbf{\Theta})}{\partial \mathbf{\Theta}} = \frac{\partial L(\mathbf{\Theta})}{\partial \mathbf{\Theta}} + \frac{\alpha}{r} \left(\frac{\partial L(\mathbf{\Theta} + \Delta\mathbf{\Theta})}{\partial \mathbf{\Theta}} - \frac{\partial L(\mathbf{\Theta})}{\partial \mathbf{\Theta}} \right) \Big|_{\Delta\mathbf{\Theta} = r \frac{\partial L / \partial \mathbf{\Theta}}{\| \partial L / \partial \mathbf{\Theta} \|_2}} = \frac{\partial L(\mathbf{\Theta})}{\partial \mathbf{\Theta}} + \alpha_1 \left(\frac{\partial L(\mathbf{\Theta} + \Delta\mathbf{\Theta})}{\partial \mathbf{\Theta}} - \frac{\partial L(\mathbf{\Theta})}{\partial \mathbf{\Theta}} \right) \Big|_{\Delta\mathbf{\Theta} = r \frac{\partial L / \partial \mathbf{\Theta}}{\| \partial L / \partial \mathbf{\Theta} \|_2}} \tag{6}$$

During the above calculation, the new hyperparameters r and α_1 were introduced. The hyperparameters r indicates how large the perturbation $\Delta\mathbf{\Theta}$ should be given to $\mathbf{\Theta}$. We directly set $r = \alpha$ during training all the ONNs and it does not influence the final training results. In this circumstance, α_1 equals 1. Therefore, the above 3 hyperparameters α, α_1, r are simplified into only α. And Equation (6) is simplified into Equation (7):

$$\frac{\partial L_1(\mathbf{\Theta})}{\partial \mathbf{\Theta}} = \frac{\partial L(\mathbf{\Theta})}{\partial \mathbf{\Theta}} + \frac{\alpha}{\alpha} \left(\frac{\partial L(\mathbf{\Theta} + \Delta\mathbf{\Theta})}{\partial \mathbf{\Theta}} - \frac{\partial L(\mathbf{\Theta})}{\partial \mathbf{\Theta}} \right) \Big|_{\Delta\mathbf{\Theta} = \alpha \frac{\partial L / \partial \mathbf{\Theta}}{\| \partial L / \partial \mathbf{\Theta} \|_2}} = \frac{\partial L(\mathbf{\Theta} + \Delta\mathbf{\Theta})}{\partial \mathbf{\Theta}} \Big|_{\Delta\mathbf{\Theta} = \alpha \frac{\partial L / \partial \mathbf{\Theta}}{\| \partial L / \partial \mathbf{\Theta} \|_2}} \tag{7}$$

From Equation (7), we can find that calculating the gradient of the new defined loss function L_1 does not require calculating the Hessian matrix, instead it can be replaced by two-steps automatic differentiation.

In the first step, the parameters $\mathbf{\Theta}$ will be updated to $\mathbf{\Theta} + \alpha \frac{\partial L / \partial \mathbf{\Theta}}{\| \partial L / \partial \mathbf{\Theta} \|_2}$, this point has the maximum loss value within the neighborhood of $\mathbf{\Theta}$. Next, the second gradient calculation is done to show the direction to reduce this maximum loss value. Finally, this two-step automatic differentiation equals minimizing Equation (1) and will effectively reduce the loss sharpness.

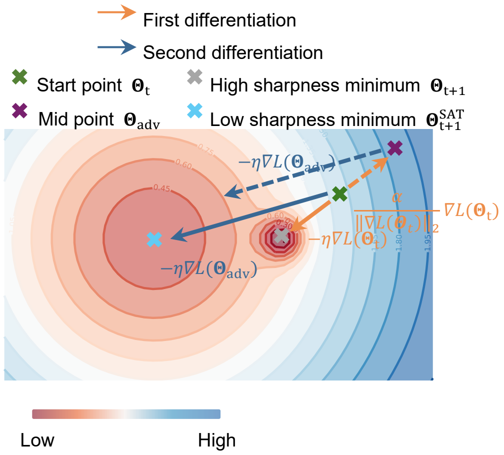

Figure S1. Schematic diagram of the proposed sharpness aware training scheme update parameter.

Pseudocode:

Input: Training set data $\mathbf{x}$.

Output: Model trained with SAT.

1. Initialize control parameters $\mathbf{\Theta}_0, t = 0$;

2. While *not converged* do

    Compute gradient $\nabla_{\mathbf{\Theta}} L(\mathbf{\Theta}_t)$;

    Update parameters $\mathbf{\Theta}_{adv} = \mathbf{\Theta}_t + \alpha \frac{\nabla_{\mathbf{\Theta}} L(\mathbf{\Theta}_t)}{\|\nabla_{\mathbf{\Theta}} L(\mathbf{\Theta}_t)\|_2}$;

    Compute gradient $\nabla_{\mathbf{\Theta}} L(\mathbf{\Theta}_{adv})$;

    Update parameters $\mathbf{\Theta}_{t+1} = \mathbf{\Theta}_t - \eta \nabla_{\mathbf{\Theta}} L(\mathbf{\Theta}_{adv})$;

    t = t + 1;

End

Return $\mathbf{\Theta}_t$

1.2. Hyperparameter α sensitivity analysis

We perform the sensitivity analysis of the defined hyperparameter α in the Microring resonator (MRR)-based computing system to show how to choose a suitable α. We use the sensitivity λ_{max} to evaluate the trained model's robustness. Small λ_{max} indicates high system robustness.

The sensitivity analysis result is shown in Figure S2, by gradually increasing the α from 0 to 100, the model's sensitivity first reduces and then increases. In contrast, the model's accuracy remains stable when α is smaller than 1 and drops significantly when we further increase the value of α. This result indicates that small perturbations can effectively increase the model's robustness while maintaining accuracy, but larger perturbations (Large sharpness penalty) would make the training totally ineffective. Based on the sensitivity analysis result, we choose the optimum value of α at 0.1, because the highest model's robustness and accuracy are achieved simultaneously at this point.

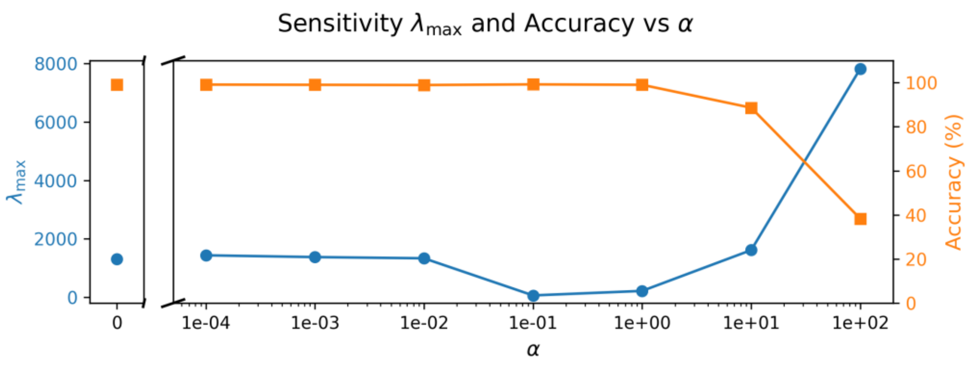

Figure S2. Sensitivity analysis result. We specifically sweep the hyperparameter α from 0 to 100, and find the neural network has the highest robustness when $\alpha = 0.1$.

2.Evaluate the trained model's robustness

The Hessian matrix reflects the curvature of loss landscape in high dimensional parameter space. The eigenvalue reflects the concave-convex property in the loss landscape. For every hessian matrix, the maximum eigenvalue corresponds to the eigenvector that has the maximum loss curvature. Therefore, the maximum eigenvalue can be used to evaluate how the loss changes with parameters, or robustness [3]. The larger the eigenvalue is, the more unstable the system is.

We employ the power iteration method to compute the top n eigenvalues and their corresponding eigenvectors of the Hessian matrix. The algorithm starts by initializing a random vector $\mathbf{v}_0$. In each iteration, we compute the product of the Hessian $\mathcal{H}$ and the current vector $\mathbf{v}_\mathrm{k}$,

$$\mathbf{v}_{\mathrm{k}+1} = \frac{\mathcal{H}\mathbf{v}_\mathrm{k}}{\|\mathcal{H}\mathbf{v}_\mathrm{k}\|_2} \tag{8}$$

Where $\mathbf{v}_{\mathrm{k}+1}$ is normalized to maintain numerical stability. The eigenvalue λ_k is approximated using the Rayleigh quotient,

$$\lambda_\mathrm{k} = \frac{\mathbf{v}_\mathrm{k}^T\mathcal{H}\mathbf{v}_\mathrm{k}}{\mathbf{v}_\mathrm{k}^T\mathbf{v}_\mathrm{k}} \tag{9}$$

The process repeats until the relative change in the eigenvalue between consecutive iterations falls below a predefined tolerance ε, i.e.,

$$\frac{|\lambda_\mathrm{k} - \lambda_{\mathrm{k}-1}|}{|\lambda_\mathrm{k}| + 1e^{-6}} < \varepsilon \tag{10}$$

To compute the subsequent eigenvalues, we apply Gram-Schmidt orthogonalization to the current vector $\mathbf{v}_\mathrm{k}$ with respect to previously computed eigenvectors to ensure that each new vector is orthogonal to the previous ones:

$$\mathbf{v}_\mathrm{k} = \mathbf{v}_\mathrm{k} - \sum_{\mathrm{i}=1}^{\mathrm{k}-1} < \mathbf{v}_\mathrm{k}, \mathbf{v}_\mathrm{i} > \mathbf{v}_\mathrm{i} \tag{11}$$

This method enables us to extract not only the largest eigenvalue but also the subsequent eigenvalues through iterative orthogonalization. The matrix-vector multiplication $\mathcal{H}\mathbf{v}_\mathrm{k}$ does not require calculating the Hessian matrix, instead it can be calculated through Equation (4) by calculating two times gradient. The evaluation process uses the open-source library 'Pyhessian' [3].

Pseudocode:

Input: Hessian matrix $\mathcal{H}$, maximum iterations ite_{max}, tolerance ε, number of eigenvalues top_n.

Output: Top n eigenvalues and eigenvectors.

1. Initialize empty lists for eigenvalues λ and eigenvectors $\mathbf{v}$;

2. Initialize computed dimension $dim = 0$;

3. While $dim < top_n$ do

Initialize random vector **v**;

Normalize **v;**

Initialize eigenvalue $\lambda = None$

For $i = 1$ to ite_{max} do

Orthogonalize **v** with respect to previously computed eigenvectors;

Compute $\mathcal{H}\mathbf{v}$, the product of the Hessian matrix and vector **v**;

Update **v** as Equation (8)

Approximate eigenvalue using Rayleigh quotient, as Equation (8);

If $\lambda = None$, then set $\lambda = \lambda_{\text{tmp}}$

Else if

$$\frac{|\lambda_{\text{k}} - \lambda_{\text{k}-1}|}{|\lambda_{\text{k}}| + 1e^{-6}} < \varepsilon$$

Then break

Update $\lambda = \lambda_{\text{tmp}}$

Append λ to eigenvalues and **v** to eigenvectors;

Increase dim

End

Return eigenvalues and eigenvectors

3.Plot the neural network 3D loss landscape

The loss landscape is drawn by perturbing along the top two Hessian eigenvectors. We calculate the top two eigenvectors of the Hessian using power iteration and perturb the model's parameters along these directions. The loss values are then evaluated over a range of perturbation magnitudes, creating a 2D grid of loss values. The 3D surface plot shows the resulting loss landscape, revealing how the model's performance changes when its parameters are shifted along the top Hessian directions.

4.MRR-based PNN experiment details

We apply three different methods to verify our method in MRR-based PNN system, and the manuscript's result is our final experiment result.

4.1. Simulation validation.

The aim of the simulation is to compare our new proposed method's effectiveness with the classic robust optimization method 'Noise-Aware Training' (NAT) [4-7], our previous work 'Optical pruning' [8] and 'Physcial-Aware Training' (PAT) [9].

NAT is widely adopted to enhance model robustness against system imperfections. NAT enhances robustness by injecting synthetic noise into the model during training, typically assuming simple additive Gaussian distributions. However, imperfections in physical systems often deviate significantly from such simplified Gaussian assumptions. These imperfections may include modeling errors and implementation-related variations such as pixel-level misalignment, fabrication errors, and temperature-induced shifts, which are non-Gaussian. As a result, NAT cannot fully capture the complex, system-specific error characteristics and thus has limitations in addressing robustness issues arising from these non-Gaussian imperfections.

In contrast, SAT makes no prior assumptions about the nature of system imperfections. Instead, it defines a clear optimization objective (i.e., sharpness minimization) that is generally applicable across different systems. By linking system robustness to the fundamental geometry of the loss landscape, SAT inherently accounts for a wide range of imperfections, including modeling inaccuracies, environmental drift, and hardware noise, without requiring explicit modeling of each. This enables SAT to optimize PNNs more accurately and robustly, even when the exact form of imperfections is unknown.

In our previous work 'optical pruning', we observed through training a neural network, when we used standard setting in backpropagation to train a neural network, although weights are distributed from -1 to 1, most weights cluster around 0. These weights are located in the noise-sensitive region of the MRR. To overcome this problem, we propose to relocate those weights from noise-sensitive region to noise-insensitive region, the off-resonance region. The loss function was modified to,

$$L_{\text{pruning}} = L(\mathbf{y}, \mathbf{y}_{\text{target}}; \mathbf{W}) + \alpha \left\| \frac{\partial \mathbf{W}}{\partial \mathbf{\Theta}} \right\|_2 \tag{12}$$

Here, $\mathbf{W}$ denotes the weights of the MRR-based PNN. $L(\mathbf{y}, \mathbf{y}_{\text{target}}; \mathbf{W})$ means optimizing weights to minimize the loss function rather than control parameters $\mathbf{\Theta}$, whose corresponding loss function is $L(\mathbf{y}, \mathbf{y}_{\text{target}}; \mathbf{\Theta})$.

Our prior work aims to train the system to a robust region. However, the method we employed was limited to relatively simple systems where such robust regions could be easily identified. For instance, in microring circuits, where each component operates almost independently, the robust region corresponds to the flat region of each individual microring's transfer function.

However, in more general physical systems, robust regions are not so easily defined. For more complex systems such as MZI networks (Section 2.3), although their behavior can be approximated by mathematical models, the robust region of the entire network is neither explicit nor easily identifiable. In even more complex scenarios, such as free-space optical systems (Section 2.4), even the system model cannot be explicitly formulated at all, making it practically impossible to identify robust regions using the approach from our previous work. Moreover, through this work, we found that even in simple systems, the robust region of individual components does not necessarily overlap with the robust region of the system as a whole.

The core contribution of SAT is that it is broadly applicable to all physical systems: SAT can automatically identify robust minima without requiring explicit knowledge of the underlying physical dynamics. Our experimental and simulation results on different systems support this generality claim. Furthermore, as illustrated in Figure 1(d) in the main text, SAT not only enhances the stability of individual components but also identifies configurations that improve the robustness of the system as a whole, thus leading to superior performance even when operating on the same hardware.

PAT is an in-situ training method designed to enable backpropagation directly on physical systems. However, it does not address resilience issues during or after training. Like other existing in-situ training approaches, PAT suffers from two key limitations: (1) The trained system is vulnerable to time-dependent degradation caused by factors such as noise, environmental disturbances, and hardware variability. (2) The trained parameters are not transferable to other systems due to device variations caused by fabrication errors.

Now, we compare these techniques with SAT on the same neural network 'LeNet-5', the detail of the network is shown in Figure S4, the network consists of two convolutional layers and three fully connected layers. The first convolutional layer takes a single-channel input and outputs six feature maps, with a 5×5 kernel and padding to preserve the input size. The second convolution layer takes the six feature maps and output sixteen, using a 5×5 kernel. Both convolutional layers are followed by batch normalization and ReLU activation to introduce nonlinearity and stabilize training. The first fully connected layer reduces the input to 120 units, the second layer reduces to 84 units, and the third layer reduces to 10 units for classification.

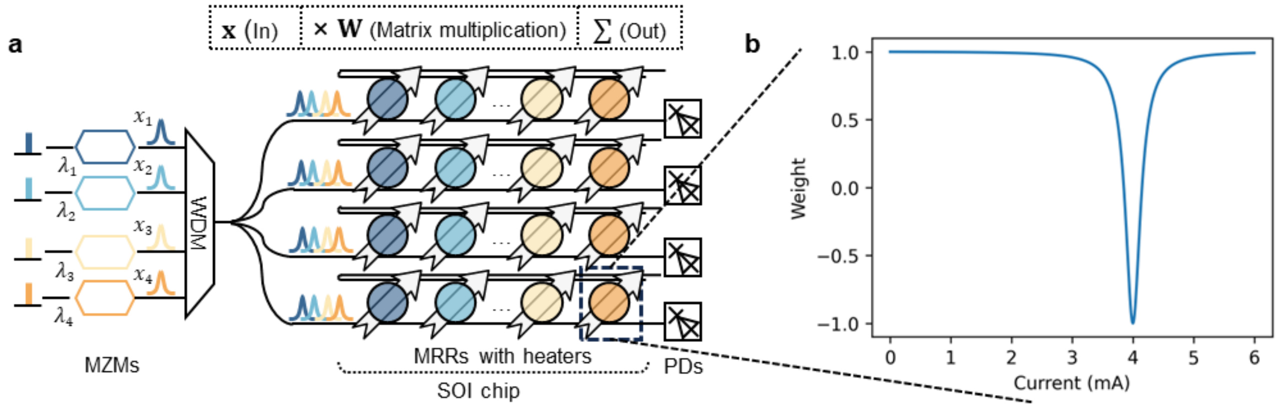

Figure S3. Illustration of MRR-based PNN control mechanism. (a) Implement matrix multiplication on MRR weight bank. (b) Measured MRR tuning curve with Lorentz curve fitting. MZM: Mach-Zehnder Modulator. WDM: Wavelength-division multiplexer. MRR: Microring resonator. PD: Photodetector. SOI: Silicon-on-insulator.

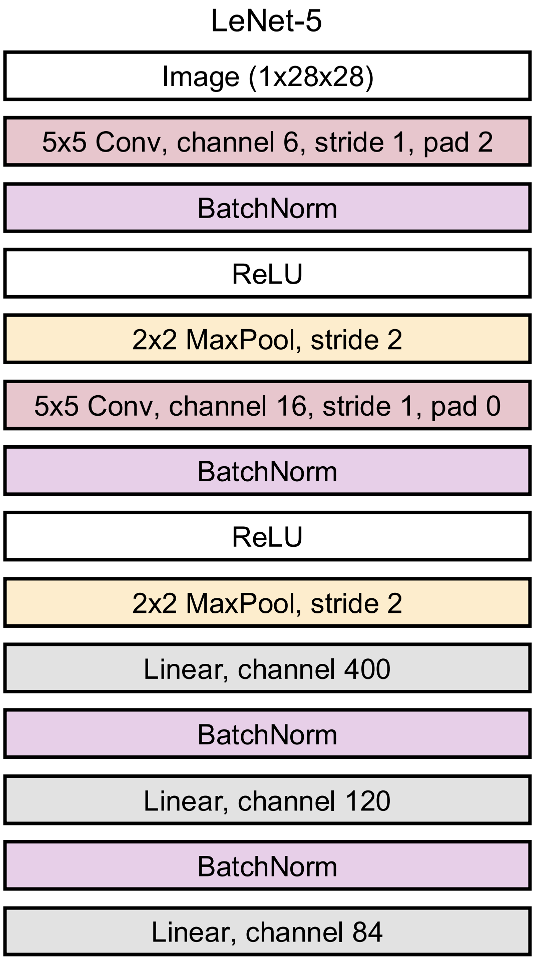

Figure S4. LeNet-5 network structure.

When using standard backpropagation (BP), NAT, optical pruning and PAT techniques, we directly optimize the weights. But when using the SAT, we make some modifications on the convolutional layers and fully connected layers to directly optimize the currents. Here, we take the convolutional layers as an example.

Example of modifying training weights to training currents (parameters):

```
class Conv2dLorentz(_ConvNd):

    def __init__(
        self,
        in_channels: int,
        out_channels: int,
        kernel_size: _size_2_t,
        stride: _size_2_t = 1,
        padding: Union[str, _size_2_t] = 0,
        dilation: _size_2_t = 1,
        groups: int = 1,
        bias: bool = True,
        padding_mode: str = 'zeros',    # TODO: refine this type
        device=None,
        dtype=None
    ) -> None:
        factory_kwargs = {'device': device, 'dtype': dtype}
        kernel_size_ = _pair(kernel_size)
        stride_ = _pair(stride)
        padding_ = padding if isinstance(padding, str) else _pair(padding)
        dilation_ = _pair(dilation)
        super().__init__(
            in_channels, out_channels, kernel_size_, stride_, padding_, dilation_,
            False, _pair(0), groups, bias, padding_mode, **factory_kwargs)

    def _conv_forward(self, input: torch.Tensor, weight: torch.Tensor, bias: Optional[torch.Tensor]):
        if self.padding_mode != 'zeros':
            return F.conv2d(F.pad(input, self._reversed_padding_repeated_twice, mode=self.padding_mode), current, bias,
self.stride, _pair(0), self.dilation, self.groups)
        return F.conv2d(input, current, bias, self.stride,
                        self.padding, self.dilation, self.groups)

    def forward(self, input: torch.Tensor) -> torch.Tensor:
        return self._conv_forward(input, lorentzian_curve(self.current, 1, 0, 0.15), self.bias)
```

By inserting the Lorentzian curve measured in the experiment, we directly modify the weights optimization to control currents optimization. The optimization hyper parameters are shown in the below Table S1,

Table S1. MRR-based PNNs simulation hyper parameters

Method	Learning rate	Epochs	Optimizer	Environment
Standard BP (In-silico)	0.001	20	SGD	Pytorch 2.1.2
NAT (In-silico)	0.05	20	SGD	Pytorch 2.1.2
Optical pruning (In-silico)	0.01	200	SGD	Pytorch 2.1.2
PAT (In-situ)	0.01	20	SGD	Pytorch 2.1.2
SAT (In-silico)	0.01	20	SGD	Pytorch 2.1.2

Note: BP-Backpropagation, NAT-Noise aware training, PAT-Physical aware training, SAT-Sharpness aware training, SGD-Stochastic gradient descent.

During the neural network training, we do not consider all these effects, but the trained model still shows high robustness against all these effects. When training with NAT, a weight noise, which follows a gaussian distribution with mean value at 0, and standard deviation at 0.3, is injected. The reason for choosing this level noise is to maximally increase the robustness while without decreasing accuracy. In the main text Figure 2j, the value of 'Standard BP' and 'SAT' are experimentally demonstrated results. The other two methods' results are estimated with simulation. In our simulation of 'PAT' method, we intentionally add experimentally measured fabrication variance to the physical forward model and the backward digital model does not contain the fabrication variance. We evaluate the model's robustness by adding sensitivity-related noise $\Delta w_{\text{noise}} = (dw/d\lambda) * \Delta\lambda$ only to the last two fully connected layers, as our experimental demonstration is conducted on the last two fully connected layers. The specific value of $dw/d\lambda$ is determined by the slope of the tuning curve as shown in Figure S3. The simulated accuracy with different temperature drift $\Delta\lambda$ is shown in Figure S5. When there is no temperature drift, the accuracy of all training methods reaches the ideal value at around 98.0%. However, the accuracy gradually decreases with the increase of temperature drift. In contrast, SAT maintains the accuracy over 90.0%. As we experimentally measured, the standard BP's accuracy is 52.0%, this corresponds to around 110pm drift in Figure S5, thus we estimate the accuracies of NAT and optical pruning accuracy are 61.0% and 73.0%, respectively. This result also shows that PAT fails to maintain performance under ambient temperature fluctuations when the thermoelectric controller (TEC) is removed and without retraining.

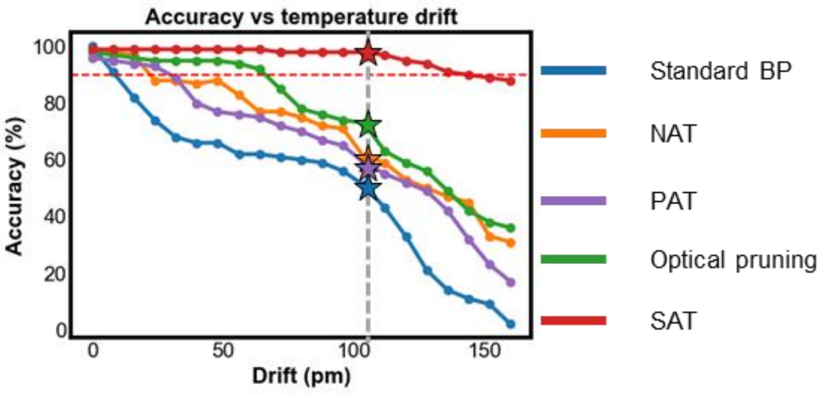

Figure S5. Simulation for estimating Optical pruning experiment accuracy without considering non-ideal modulation effects. BP: Backpropagation. NAT: Noise aware training. PAT: Physical aware training. SAT: Sharpness aware training.

4.2. Single MRR experimental validation.

After getting the preliminary results through simulation, we further conduct a simple experiment to validate the simulation results. The MRR weight bank is fabricated in the commercial multi-project wafer (MPW) process from the Applied Nanotools (ANT). The radii for each MRR is around 20 µm, and we intentionally introduce a slight 10 nm radius difference to avoid resonance collision. The experimental setup is shown in Figure S6.

The detailed experimental step goes by:

i. Set the laser wavelength to off-resonance region. The measured optical power P_{ref} is -12dBm;

ii. Sweep actuating voltage 0~2V, then measure the corresponding optical power P_{mea}, and get the voltage-weight relationship or look up table (LUT). Now the weight tuning range is [0,1], to achieve negative weight, we manually scale the weight as;

$$w_{[-1,1]} = 2w_{[0,1]} - 1 \tag{13}$$

iii. According to the target weight, actuate the corresponding voltage;

iv. Measure the weight and calculate the weight error.

During the experiment, we randomly sample 500 weights from the neural network weight distributions trained by different methods independently. Then we implement the weights according to the LUT and calculate the weight errors. Next, we add noises to the trained neural networks by randomly sampling weight errors from the statistical distribution of weight errors obtained from experiments and perform inference on computer. This validation method's correctness was verified in our previous work.

We intentionally change the temperature and actuate random voltage on MRR 12 and MRR 14 to emulate the temperature fluctuation, electrical and thermal crosstalk. The experimental results are shown in Table S2 and Table S3. From Table S2, we can see pruning achieves the lowest weight error compared with Standard BP and SAT, this result is consistent with our previous work. In fact, the error is bigger than our previous result because the spectrum of this MRR weight bank is not as flat as our previously used MRR weight bank. However, when we compare the inference accuracy, SAT remains ideal accuracy while other two methods have accuracy reduction. These results are consistent with previous simulation results. They show that by considering both the component stability and system stability, SAT can outperform Pruning. The results also show that small component error does not definitely result in small system error.

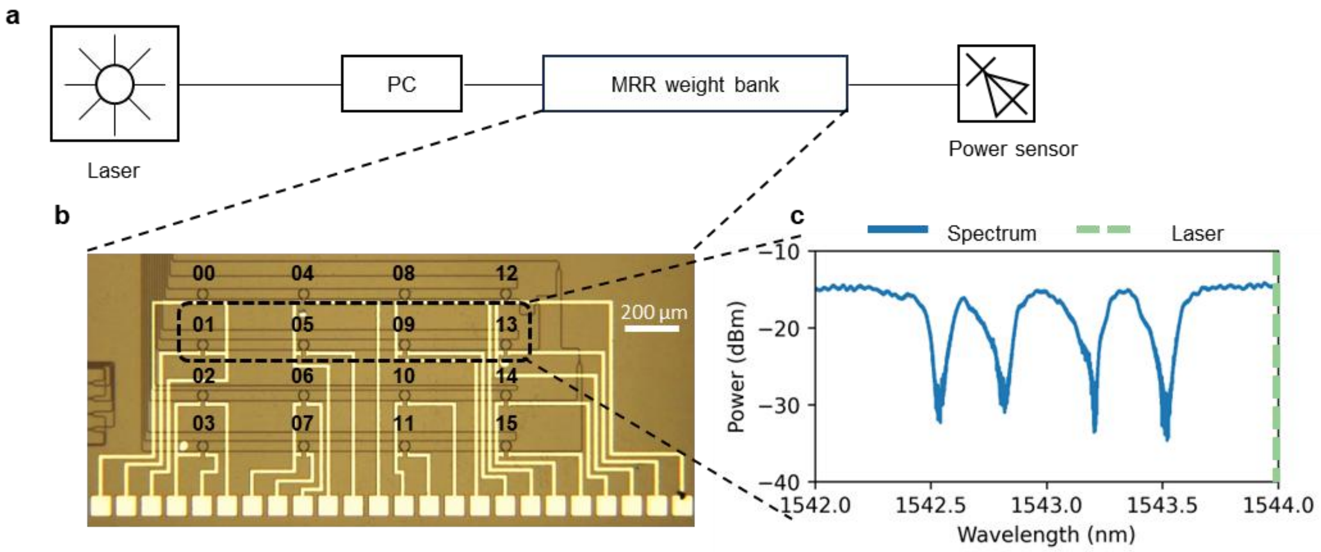

Figure S6. Single MRR experimental validation experimental setup and measured spectrum. (a) Experimental setup. (b) Fabricated MRR weight bank's picture. (c) Measured MRR weight bank spectrum. PC: Polarization. MRR: Microring resonator.

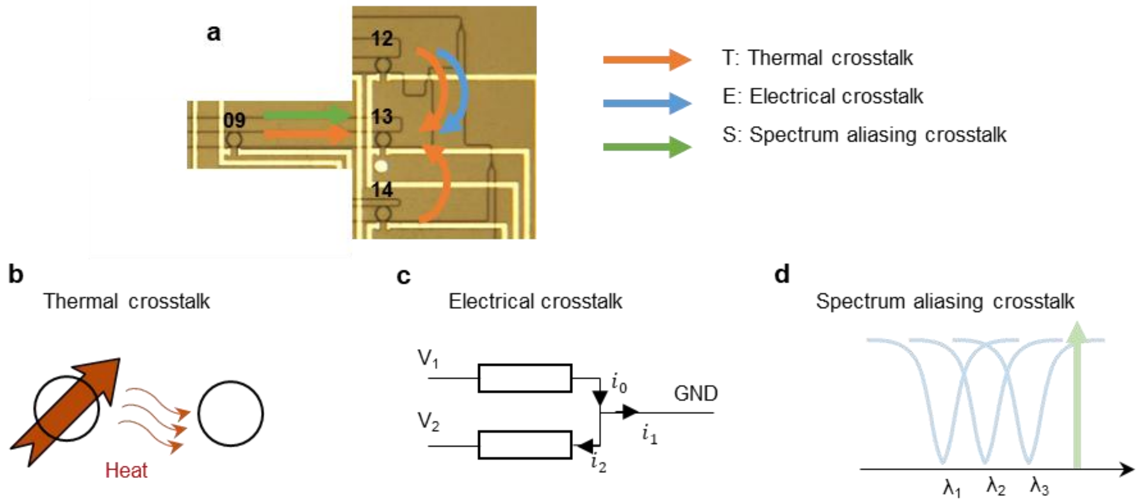

Figure S7. Single MRR experimental validation imperfections analysis. (a) Illustration of how different kinds of crosstalk influence the MRR. (b) Thermal crosstalk illustration figure. (c) Electrical crosstalk illustration figure. (d) Spectrum aliasing crosstalk illustration figure.

Table S2. Experimental measured Root-mean-square-error for different training methods.

RMSE	Standard BP	Pruning	SAT
w/o (> 7 bit)	0.010	0.015	0.007
Crosstalk 0.5V	0.177	**0.046**	**0.110**
Drift +0.1 ℃	0.126	**0.079**	**0.093**

Note: RMSE-Root mean square error, BP-Backpropagation, SAT-Sharpness aware training. 'w/o' represents without thermal crosstalk, electrical crosstalk and temperature drift.

Table S3. Experimental measured accuracy for different training methods.

RMSE	Standard BP	Pruning	SAT
w/o (> 7 bit)	98.78%	97.81%	98.86%
Crosstalk 0.5V	16.99%	**92.56%**	**98.61%**
Drift +0.1 ℃	16.78%	**86.62%**	**98.76%**

Note: BP-Backpropagation, SAT-Sharpness aware training. 'w/o' represents without thermal crosstalk, electrical crosstalk and temperature drift.

4.3. Large scale MRR experimental validation.

After having the validation results in single MRR, we further perform the neural network inference on our chip to verify our approach's improvement in accuracy. In our experiments, we perform the final two layers inference on our MRR weight bank. We decompose the large matrix multiplication into small matrix multiplication, and the method is the same as our previous work [8]. The experimental setup and details are shown in Figure S8. We select four MRRs labeled '00', '03', '12', and '15' for the experiment. Initially, currents are applied to align the resonance wavelengths: 1542.7 nm for '00' & '03', and 1544.8 nm for '12' & '15'. A Thermoelectric controller (TEC) module controls temperature stabilization or variation. The laser wavelengths are set to 1543.0 nm and 1545.0 nm. The modulated lights were combined via a 50:50 coupler and amplified by an EDFA to around 12 dBm before coupling into the chip, with a coupling loss of ~14 dBm. Two output channels are detected by PD at the through port. For matrix multiplication, negative weights are mapped to the tuning range [0,1].

Here we show how to map the negative weights to the tuning range [0,1].

$$\begin{bmatrix} S_{\text{path1}}(t) \\ S_{\text{path1}}(t) \end{bmatrix} = \begin{bmatrix} \hat{w}_{11} & \hat{w}_{12} \\ \hat{w}_{21} & \hat{w}_{22} \end{bmatrix} \begin{bmatrix} S_{\text{ch1}}(t) \\ S_{\text{ch2}}(t) \end{bmatrix} = 2 \begin{bmatrix} \frac{\hat{w}_{11}+1}{2} & \frac{\hat{w}_{12}+1}{2} \\ \frac{\hat{w}_{21}+1}{2} & \frac{\hat{w}_{22}+1}{2} \end{bmatrix} \begin{bmatrix} S_{\text{ch1}}(t) \\ S_{\text{ch2}}(t) \end{bmatrix} - \begin{bmatrix} S_{\text{ch1}}(t) + S_{\text{ch2}}(t) \\ S_{\text{ch1}}(t) + S_{\text{ch2}}(t) \end{bmatrix} \tag{14}$$

We first scale the weight from [-1,1] region to [0,1] region, then perform the matrix multiplication of $\begin{bmatrix} \frac{\hat{w}_{11}+1}{2} & \frac{\hat{w}_{12}+1}{2} \\ \frac{\hat{w}_{21}+1}{2} & \frac{\hat{w}_{22}+1}{2} \end{bmatrix} \begin{bmatrix} S_{\text{ch1}}(t) \\ S_{\text{ch2}}(t) \end{bmatrix}$. Here, $S_{\text{path1}}(t)$ and $S_{\text{path2}}(t)$ denote the matrix-vector multiplication results measured by the PDs in two different paths. The weights that are implemented on MRR '00','03','12' and '15' are $\frac{\hat{w}_{11}+1}{2}$, $\frac{\hat{w}_{21}+1}{2}$, $\frac{\hat{w}_{12}+1}{2}$, $\frac{\hat{w}_{22}+1}{2}$. $S_{\text{ch1}}(t)$ and $S_{\text{ch2}}(t)$ are modulated signals. They are normalized to [0,1] before being modulated on different light wavelengths. Next, we manually minus the input signal and scale the result to match the ideal value. The modulated signal waveforms are shown in Figure S8(b). The average root-mean-square-error (RMSE) is around 0.05.

During the experiments, we also characterize the wavelength-dependent gain in the EDFA and non-ideal modulations.

(1) Characterization of wavelength-dependent gain in the EDFA

The characterization setup is shown in Figure.S9. By sweeping the wavelength, we measure the amplified light intensity from the power sensor. The result shows the EDFA has a gain fluctuation at around 1dB from 1530nm to 1570nm.

(2) Characterization of the non-ideal modulations

We first characterize the single channel non-ideal modulation. The experimental setup is shown in Figure S10. We modulate a ramp signal on the modulator and measure the detected signal peak-to-peak voltage Vpp. We do two independent tests on both modulators, and the test results both show the bias point is time changing, as depicted in Figure S10. We calculate the RMSE is around 0.05. Next, we combine the two channels and do the characterization again. The characterization experimental setup is the same as Figure S8(a). Before, we independently tune the light power to balance the EDFA wavelength-dependent gain. We set all MRRs to the off-resonance region, then we modulate two same frequency ramp signals on two MZMs, and we measure the signal's peak-to-peak value from the scope. We do three independent tests. The experimental results are shown in Figure S11. During the test, we iteratively transform three steps **(i)** Turn on CH1 signal, turn off CH2 signal, measure Vpp, label 'ch1'; **(ii)** Turn off CH1 signal, turn on CH2 signal, measure Vpp, label 'ch2'; **(iii)** Turn on CH1 signal and CH2 signal, measure Vpp, label 'sum'. The results (Figure S11) show the non-ideal modulation influences the calculation results. The RMSE is around 0.07, which is at the same level as single channel characterization.

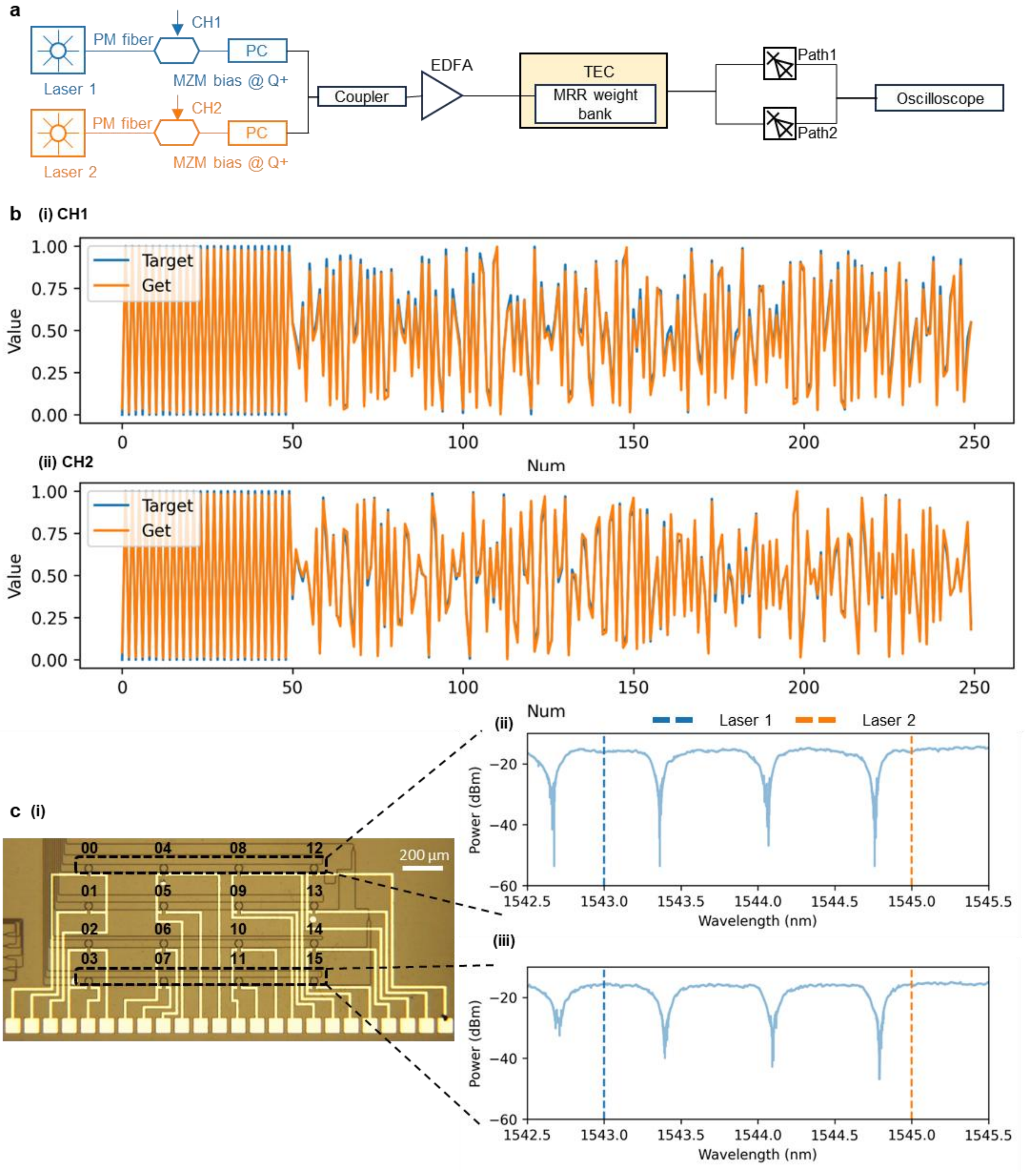

Figure S8. Large scale MRR experimental validation imperfections analysis. (a) Schematic diagram of the experimental setup. (b-i) CH1 Experimental measured waveforms. (b-ii) CH2 Experimental measured waveforms. (c-i) Fabricated MRR weight bank's picture. (c-ii) Measured spectrum of MRR '00-04-08-12'. (c-iii) Measured spectrum of MRR '03-07-11-15'. PM: Polarization maintain. MZM: Mach-Zehnder Modulator. PC: Polarization controller. EDFA: Erbium-doped fiber amplifier. TEC: Thermoelectric controller. MRR: Microring resonator.

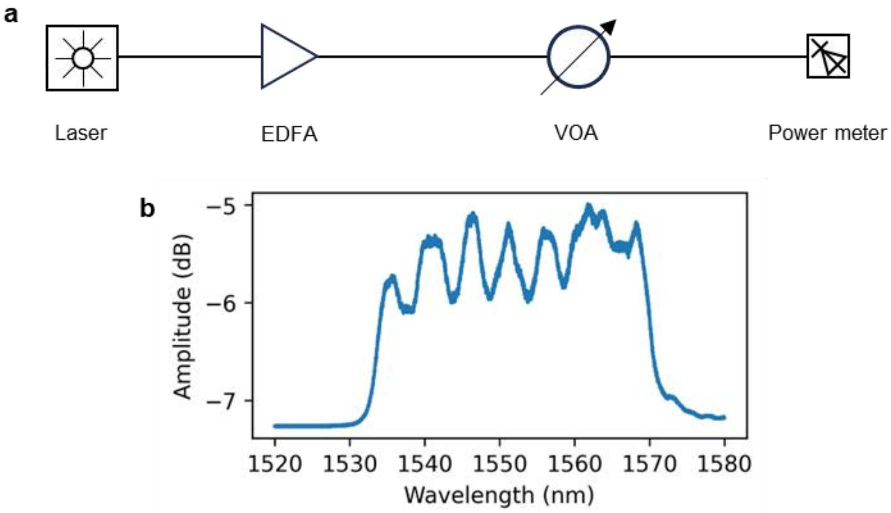

Figure S9. Characterization results of EDFA gain competition. (a) Characterization experimental setup. (b) Measured EDFA gain spectrum. EDFA: Erbium-doped fiber amplifier. VOA: Variable optical attenuator.

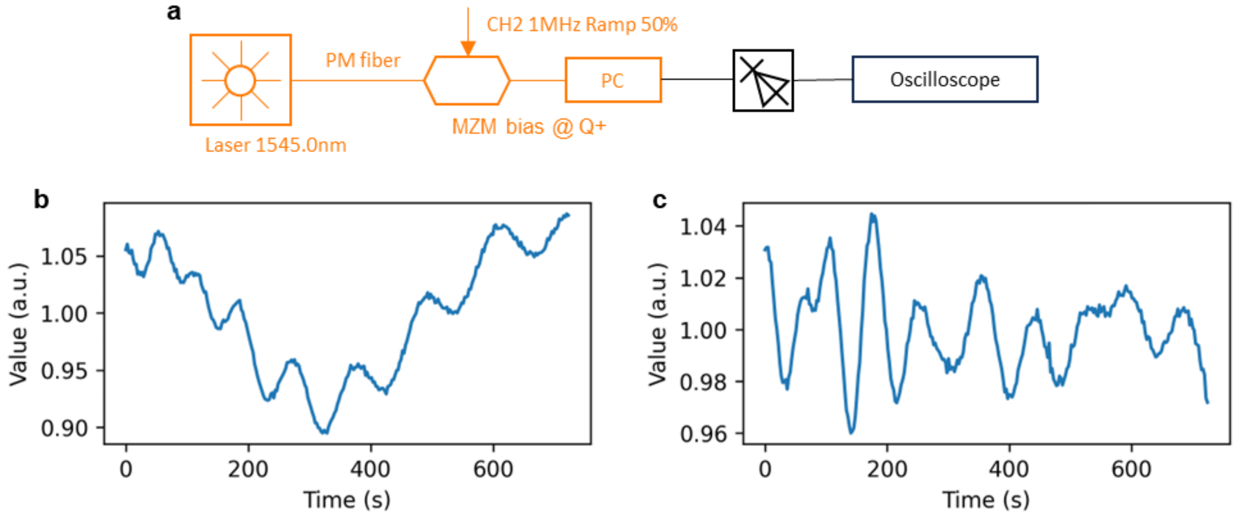

Figure S10. Characterization results of single channel non-ideal modulations. (a) Experimental setup. (b) The light intensity changes after passing through the modulator, first experiment. (c) The light intensity changes after passing through the modulator, second experiment.

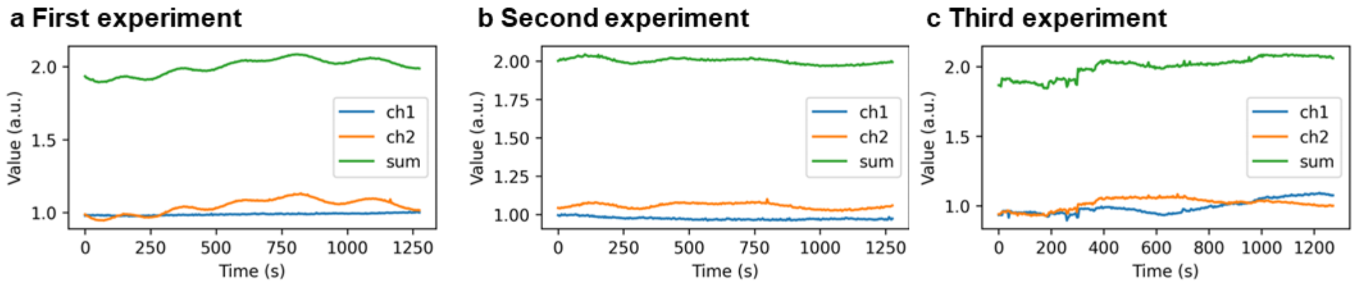

Figure S11. Characterization results of two channels non-ideal modulations. (a) The light intensity changes after passing through the modulator 'ch1' and 'ch2', as well as the summed light intensity change, first experiment. (b) The light intensity changes after passing through the modulator 'ch1' and 'ch2', as well as the summed light intensity change, second experiment. (c) The light intensity changes after passing through the modulator 'ch1' and 'ch2', as well as the summed light intensity change, third experiment.

4.4. Extend the verification to CIFAR-10 dataset.

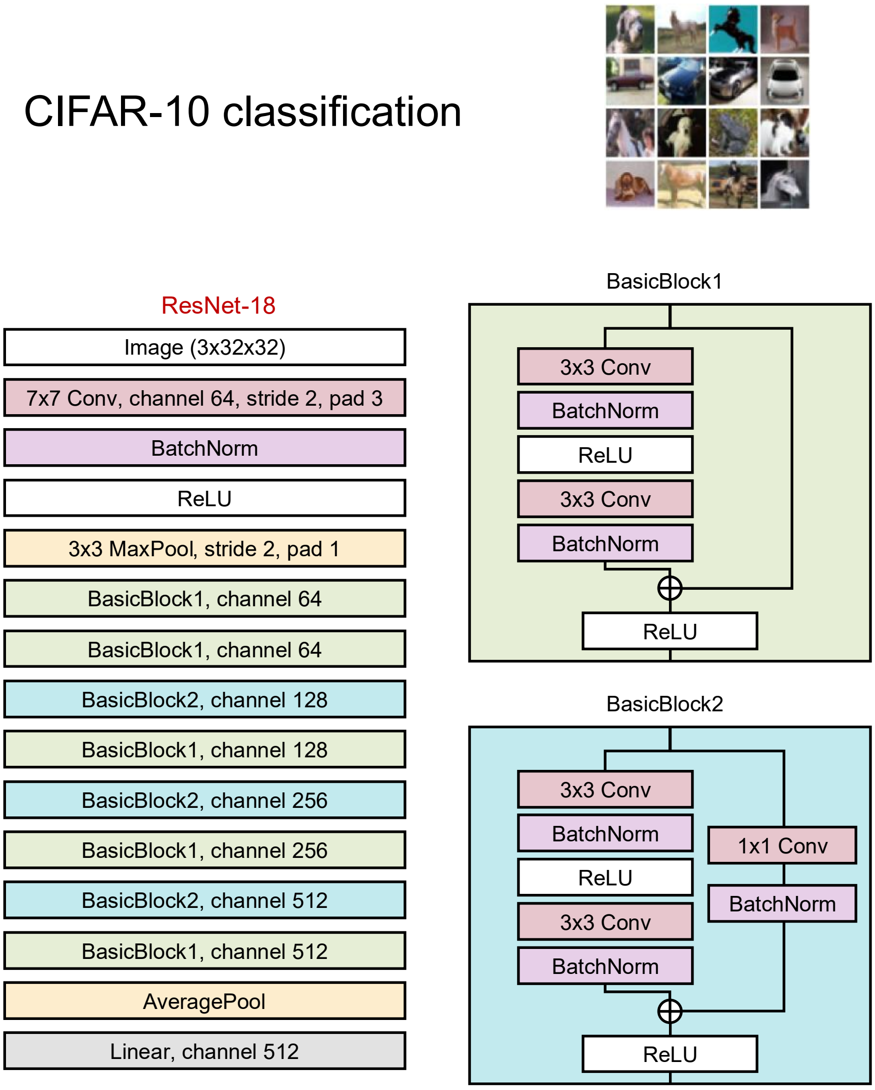

Figure S12. ResNet18 for CIFAR-10 image classification.

Table S4. MRR-based PNNs simulation hyper parameters

Method	Learning rate	Epochs	Optimizer	Environment
Standard BP	0.001	100	SGD	Pytorch 2.1.2
SAT	0.001	500	SGD	Pytorch 2.1.2

Note: BP-Backpropagation, SAT-Sharpness aware training, SGD-Stochastic gradient descent.

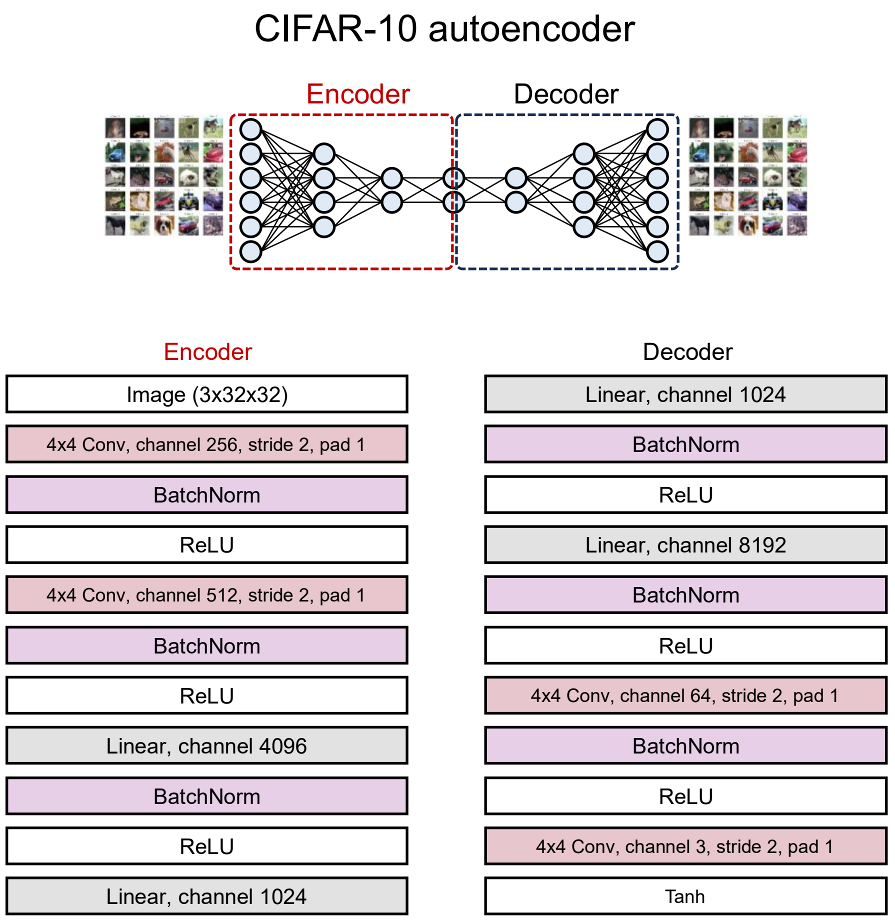

Figure S13. Autoencoder for CIFAR-10 image compression and reconstruction.

Table S5. MRR-based PNNs simulation hyper parameters

Method	Learning rate	Epochs	Optimizer	Environment
Standard BP	0.001	300	Adam	Pytorch 2.1.2
SAT	0.005	300	Adam	Pytorch 2.1.2

Note: BP-Backpropagation, SAT-Sharpness aware training.

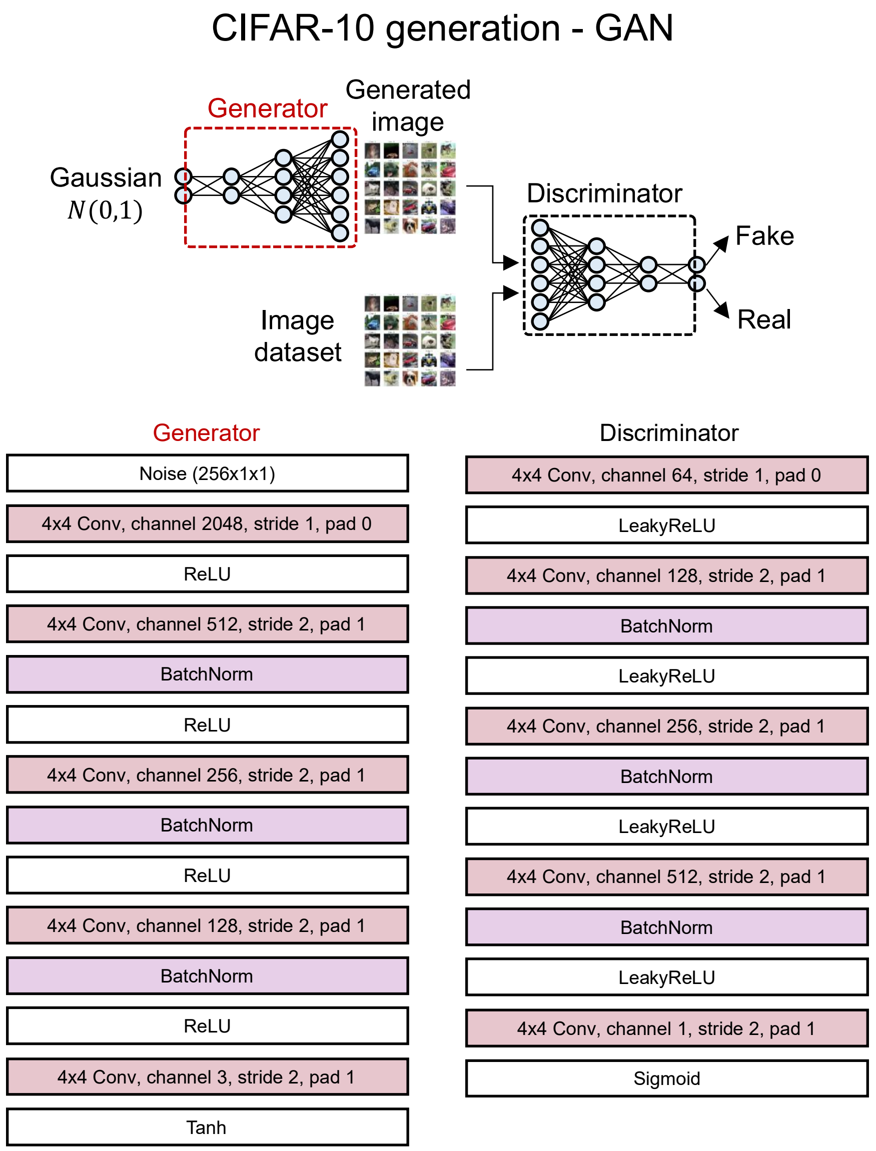

Figure S14. GAN for CIFAR-10 image generation. GAN-Generative adversarial network.

Table S6. MRR-based PNNs simulation hyper parameters

Method	Learning rate	Epochs	Optimizer	Environment
Standard BP	0.0005	1500	Adam	Pytorch 2.1.2
SAT	0.0005	1500	Adam	Pytorch 2.1.2

Note: BP-Backpropagation, SAT-Sharpness aware training.

The network structures for different tasks are shown in Figure S12-14, and the corresponding training hyper parameters are shown in Table S4-6. Specifically, for the image classification, we choose the whole 10 categories dataset (airplane, automobile, bird, cat, deer, dog, frog, horse, ship and truck). In terms of the image compression and reconstruction, we select the 4 categories dataset (horse, frog, dog and automobile). For the image generation, we apply only the 'horse' category. In all the above tasks, we evaluate the robustness of the networks by adding the sensitivity-related noise $\Delta w_{\text{noise}} = (dw/d\lambda) * a * \Delta T$ only to the layers that are implemented in the MRR-based PNN. Here $dw/d\lambda$ represents the slope of the MRR spectrum, a denotes the thermal sensitivity of the MRR and we set it at 0.08nm/K, ΔT is the temperature change. In the CIFAR-10 classification task, the ResNet18 network is entirely implemented in MRR-based PNN. In the CIFAR-10 compression and reconstruction task, the encoder part is implemented in MRR-based PNN. And in the CIFAR-10 generation task, the generator part is implemented in MRR-based PNN.

5.MZI-based PNNs simulation details

We build a digital-optical hybrid network to conduct simulations on MZI-based PNNs [10]. The neural network's structure is shown in Figure S15. We slightly modify the classic LeNet-5 architecture's final fully connected layer to the 64×64 MZI mesh, followed by a square detection layer. It begins with two convolutional layers followed by max-pooling operations. The first convolutional layer takes a single-channel input and outputs six feature maps, with a 5×5 kernel and padding to preserve the input size. The second convolutional layer takes the six feature maps and outputs sixteen, using a 5×5 kernel. Both convolutional layers are followed by batch normalization and ReLU activation to introduce nonlinearity and stabilize training. After the convolutional layers, the feature maps are flattened into a one-dimensional vector and passed through two fully connected layers. The first fully connected layer reduces the input to 120 units, and the second layer further reduces it to 64 units. Batch normalization is applied after the first fully connected layer to improve training performance. The output of the second fully connected layer is then transformed into a complex tensor by stacking it with a zero-filled tensor, which prepares the data for the optical MZI mesh layer. This layer performs unitary matrix multiplications and allows for the quantitative inclusion of phase and beam splitting errors. The final output is passed through a square detection layer, which computes the squared norm of the output.

The 64×64 MZI mesh is Clements structure [11]. The mesh is constructed with multiple MZIs formed as the rectangular grid. The basic building block 2×2 MZI consists of two 50:50 beamsplitters and two phase shifters. The transmission matrix of a 2×2 MZI can be expressed as,

$$T = \frac{1}{2}\begin{bmatrix} 1 & i \\ i & 1 \end{bmatrix}\begin{bmatrix} e^{i\theta} & 0 \\ 0 & 1 \end{bmatrix}\begin{bmatrix} 1 & i \\ i & 1 \end{bmatrix}\begin{bmatrix} e^{i\varphi} & 0 \\ 0 & 1 \end{bmatrix} = ie^{\frac{i\theta}{2}}\begin{bmatrix} e^{i\varphi}\sin\left(\frac{\theta}{2}\right) & \cos\left(\frac{\theta}{2}\right) \\ e^{i\varphi}\cos\left(\frac{\theta}{2}\right) & -\sin\left(\frac{\theta}{2}\right) \end{bmatrix} \tag{15}$$

Where θ, φ are single-mode phase shifts on the top arm.
For the ideal beam splitter, the transmission matrix is,

$$\frac{1}{\sqrt{2}}\begin{bmatrix} 1 & i \\ i & 1 \end{bmatrix} = \begin{bmatrix} \cos\left(\frac{\pi}{4}\right) & i\sin\left(\frac{\pi}{4}\right) \\ i\sin\left(\frac{\pi}{4}\right) & \cos\left(\frac{\pi}{4}\right) \end{bmatrix} \tag{16}$$

However, the manufacturing error will introduce beamsplitter errors and phase shifter errors in MZIs.
The beamsplitter error will make the transmission matrix become,

$$\begin{bmatrix} \cos\left(\frac{\pi}{4}+\alpha\right) & i\sin\left(\frac{\pi}{4}+\alpha\right) \\ i\sin\left(\frac{\pi}{4}+\alpha\right) & \cos\left(\frac{\pi}{4}+\alpha\right) \end{bmatrix} \tag{17}$$

During our simulation, we define the splitter ratio error ε and rewrite the transmission matrix as,

$$\frac{1}{\sqrt{2}}\begin{bmatrix} \sqrt{1+\varepsilon} & i\sqrt{1-\varepsilon} \\ i\sqrt{1-\varepsilon} & 1\sqrt{1+\varepsilon} \end{bmatrix} \tag{18}$$

The phase shifter error will directly lead to uncertainty on the phase shifters' values.

Before the simulation, we random phase error and splitting error, and use them as the static errors for the neural network inference. Specifically, we test 6 different training methods, the training flow is shown in Figure S16, Figure S17 [6] and Figure S18. Here we provide detailed training hyperparameters for different training methods, as listed in Table S7.

Table S7. Training hyperparameters for different training methods.

Method	Learning rate	Epochs	Environment
Standard BP (In-silico)	0.1	40	Pytorch 2.1.2
NAT (In-silico) [4-7]	0.1	40	Pytorch 2.1.2
PAT (In-situ) [9]	0.1	40	Pytorch 2.1.2
DAT (In-situ) [10]	0.1	60	Pytorch 2.1.2
SAT (In-silico)	0.1	40	Pytorch 2.1.2
SAT (In-situ)	0.1	40	Pytorch 2.1.2

Note: BP-Backpropagation, NAT-Noise aware training, PAT-Physical aware training, DAT-Dual adaptive training, SAT-Sharpness aware training.

During training with NAT, the noise we add to the phase parameter follows a gaussian distribution with mean value at 0, and standard deviation at 0.01 rad. The aim is to maximally increase the robustness while without decreasing the accuracy.

The first 30 epochs for DAT process are the same as PAT, and system error prediction network (SEPN) is trained to reduce the output difference between the physical system and the ideal system, but it is not used for backpropagation. In the subsequent 30 training epochs, SEPN training stops, and it assists the ideal model to provide accurate gradients. The learning rate for training the SEPN is 0.001 for constant.

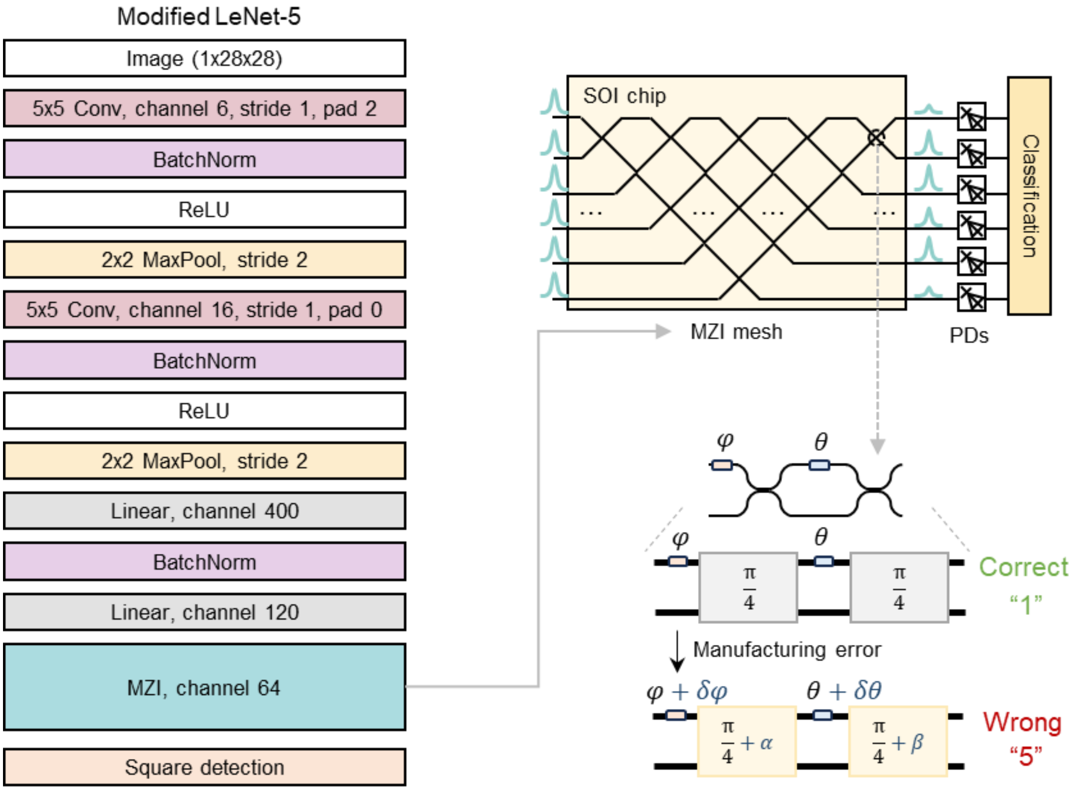

Figure S15. Modified LeNet-5 network structure.

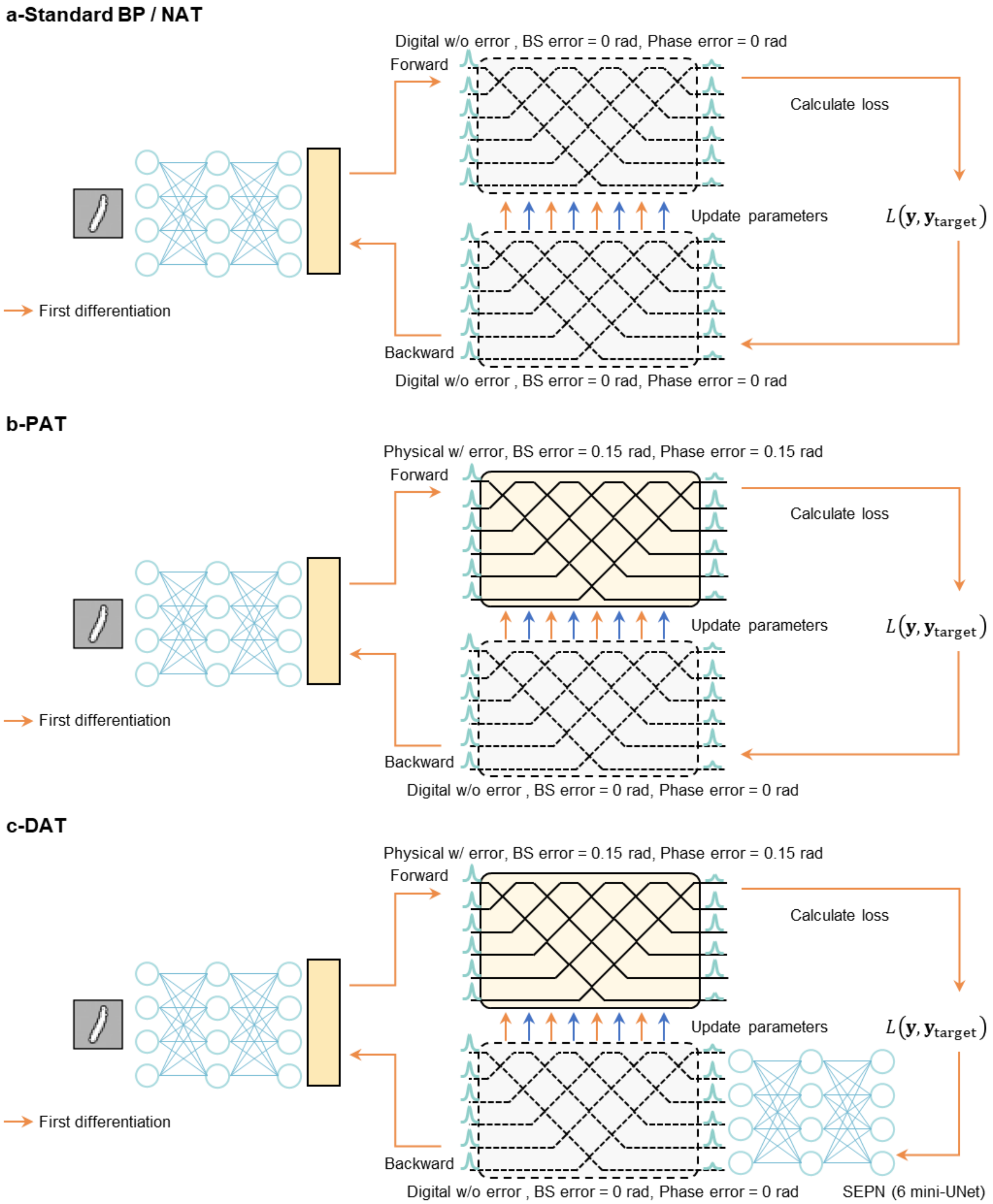

Figure S16. Referenced training methods. (a) Standard BP/NAT. (b) PAT. (c) DAT. BP: Backpropagation. BS: Beam-splitter. NAT: Noise aware training. PAT: Physical aware training. DAT: Dual adaptive training. SEPN: System error prediction error.

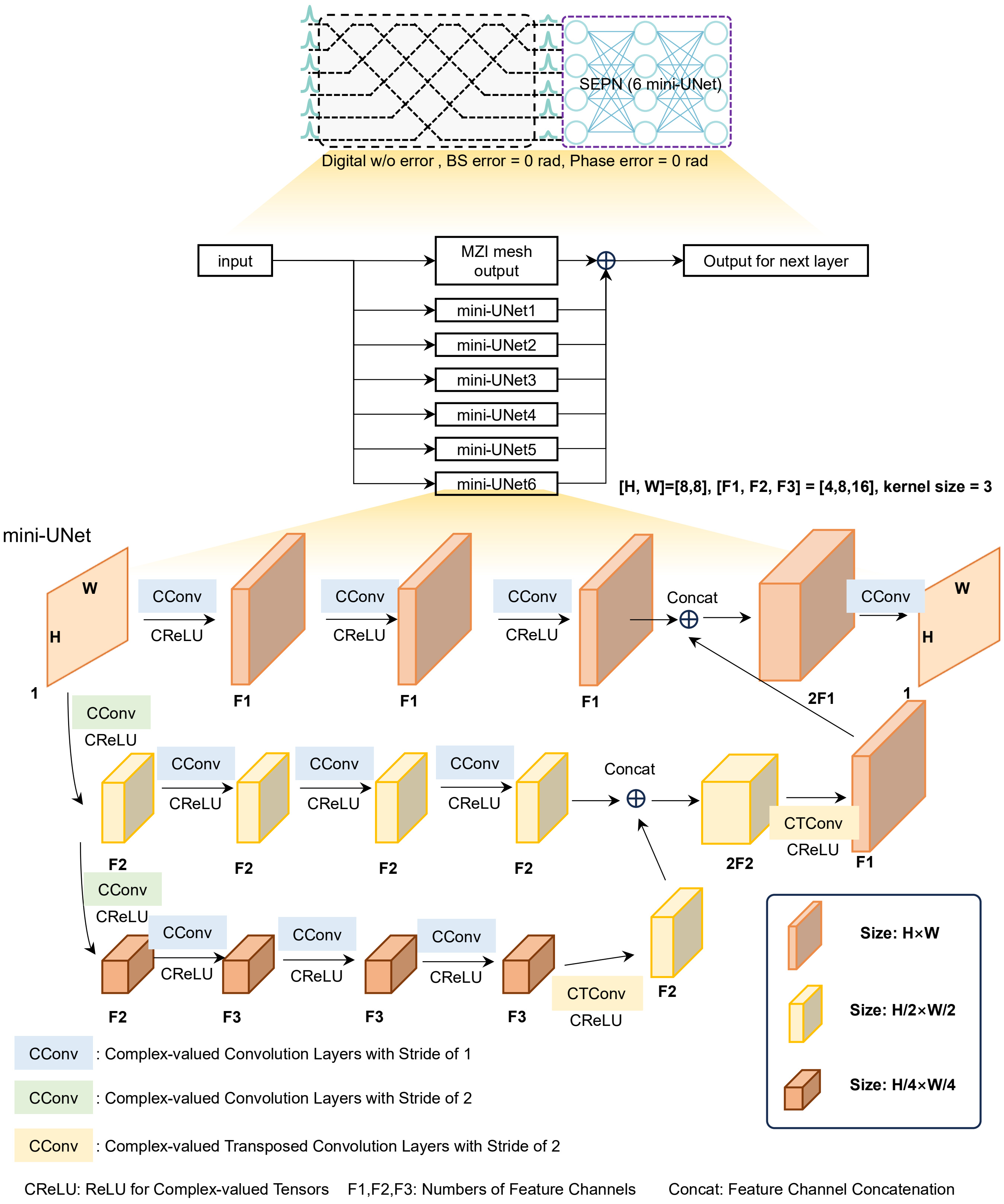

Figure S17. Detailed network structure of DAT with SEPN. SEPN: System error prediction error [10].

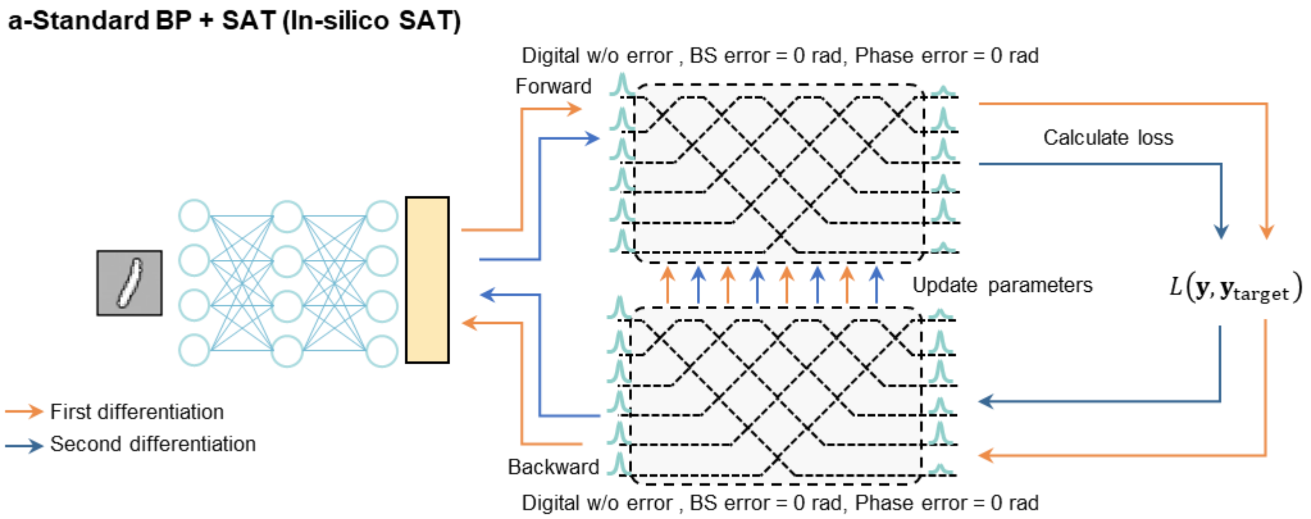

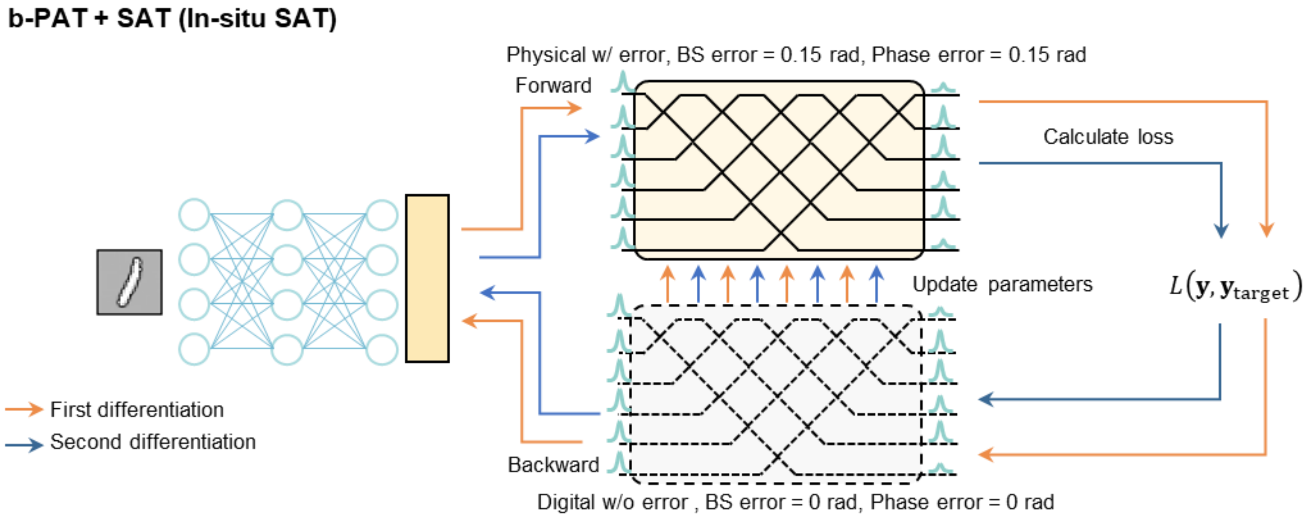

Figure S18. Implement proposed method on (a) in-silico training and (b) in-situ training. BP: Backpropagation. BS: Beam-splitter. SAT: Sharpness aware training.

6.Diffractive optics-based NNs experiment details

6.1. Experimental setup details and basic characterization

Our experimental setup is similar to [12]. We use an organic light-emitting diode (OLED, Sony ECX335S) as the light source for encoding input vectors. The OLED has R (Red)/G (Green)/B (Blue) three channels, and we only use the green channel. The corresponding emission spectrum centers around 525 nm. The OLED display consists of an array of 1920 × 1080 pixels, and its refreshing rate is 60Hz. We develop custom Python scripts to control the OLED and load the bitmap images onto the OLED display from a control computer. The OLED can produce 256 distinct brightness levels corresponding to 8 bits resolution. During the matrix-vector multiplication computations, the input vector is reshaped into a 2D block and displayed as an image on the OLED screen. We duplicate the vector block on the OLED display to exploit the parallelism of this system. An intensity-modulation module is implemented using a phase-only reflective liquid-crystal-on-silicon spatial light modulator (LCOS-SLM, HDSLM80R Plus, UPO labs) paired with a polarization beam splitter and a half-wave plate in a double-pass configuration. Before using the SLM, we characterize the intensity gamma curve to map the SLM pixel values to specific transmission percentages. A zoom-lens microscope (BYH0330, Inseinlifung) magnifies the OLED pixel image by approximately 1.026× to match the pixel pitch of the SLM. The image of each OLED pixel is diffraction-limited to a spot size of ~5 μm (constrained by the microscope 1), which is smaller than the SLM pixel size of 8 μm, ensuring no crosstalk between

neighboring pixels. Pixel-to-pixel alignment is achieved for one million pixels. The resulting optical intensity field is imaged onto the camera plane using a second microscope (SHL-0745C, Shunhuali). The CMOS camera (MV-SUF89OGC/M, Mindvision) captures the intensity of the modulated light field as an image. This image is divided into regions of interest (ROIs), with each ROI representing the element-wise product of the two vectors. By summing the pixels within each ROI, we obtain the total photon counts, corresponding to the dot product between the two vectors.

Here, we provide the details of our experimental setup calibration,

(1) Characterization of OLED and Gamma curve

The gamma curves of OLED and SLM describe the nonlinear relationship between the input signal and the light brightness and transmission, respectively. For OLED displays, the gamma curve illustrates how the input signal, typically the pixel intensity or voltage, translates into brightness. And in an SLM, the gamma curve reflects how the input signal controls the modulated light transmission. This relationship is nonlinear and often follows an exponential form:

$$L = V^{\gamma} \tag{19}$$

Where L is the brightness for OLED and transmission for SLM, V is the control voltage, and γ is the gamma value.
We measure the gamma curves by sweeping voltages of OLED and SLM, we then measure the light intensity at the camera. The measured curves are shown in Figure S19 and S20.

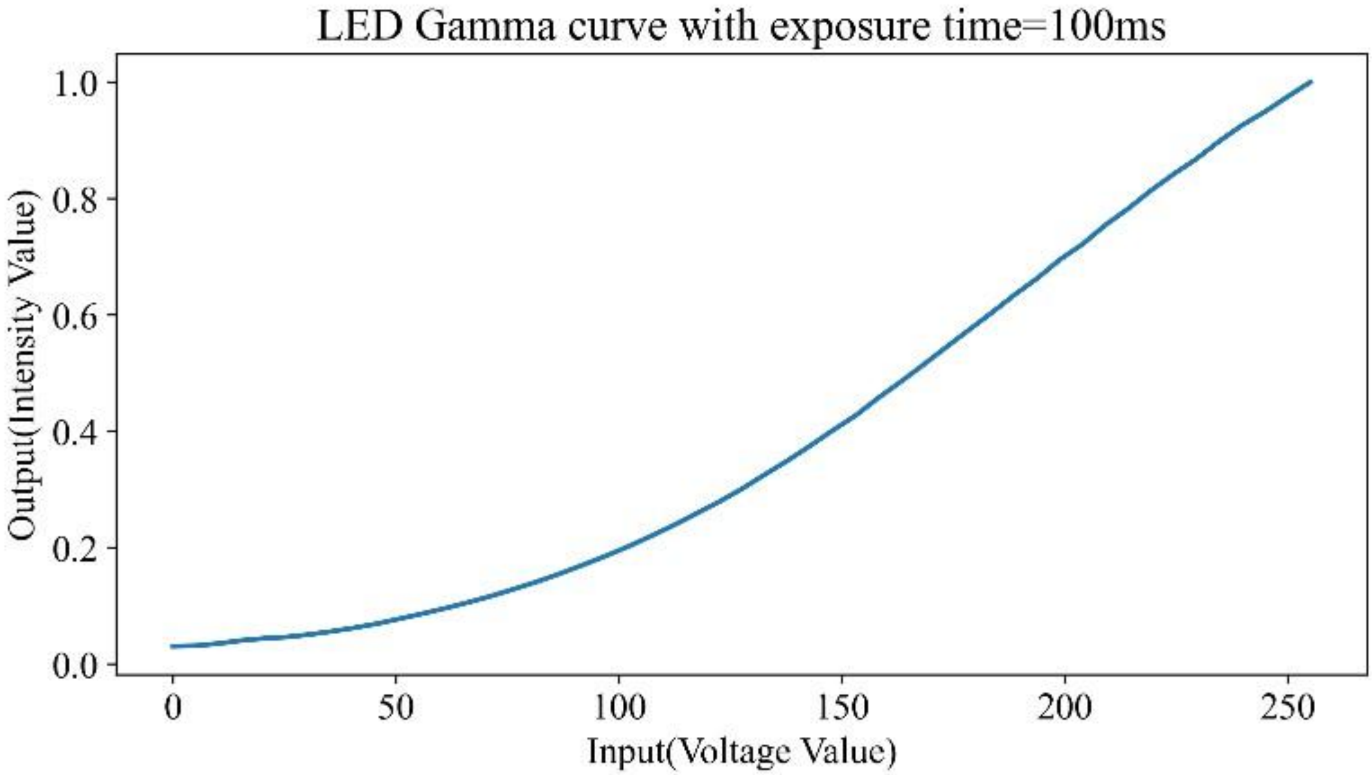

Figure S19. Measured OLED gamma curve.

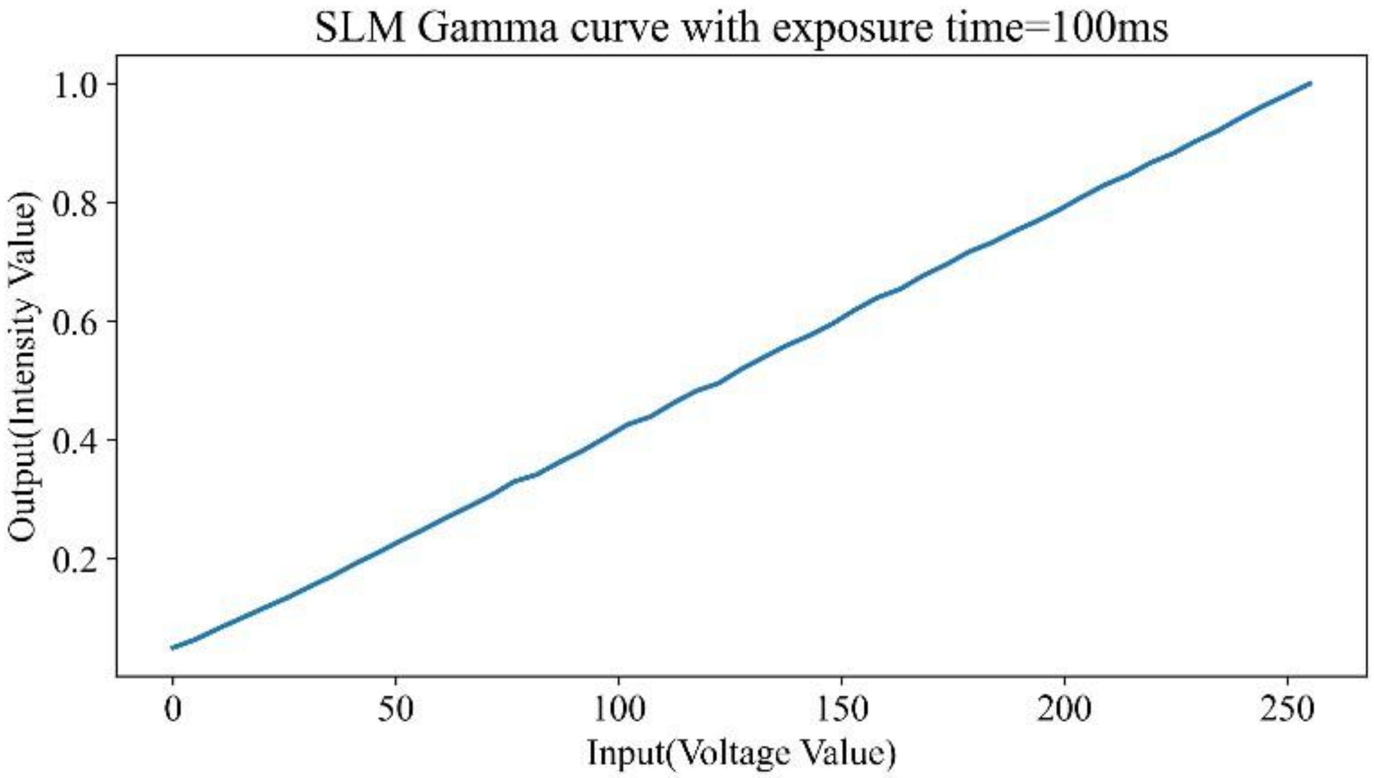

Figure S20. Measured SLM gramma curve.

(2) Align the optical imaging system

The purpose of pixel-to-pixel alignment is to maximumly alleviate every pixel to perform optical matrix multiplication. The alignment follows three criteria,

1. The OLED display must be imaged onto the SLM with precise magnification to match the pixel pitches of both devices;
2. The imaging resolution must ensure each OLED pixel image on the SLM is no larger than an SLM pixel to avoid crosstalk;
3. Each OLED pixel image must align with the corresponding SLM pixel, requiring fine adjustments in translation, rotation, pitch, and yaw of the devices.

The detailed alignment steps include,

1. Display identical images of the same size on both the OLED display and the SLM. On the OLED display, bright pixels correspond to fully transmitting pixels on the SLM, while dark pixels correspond to zero transmission pixels. Here the SLM acts as a mask, where the light-transmitting areas match the bright image on the OLED display in shape and size.
2. Check whether the image on the camera remains unclipped as the image on the OLED display. The reason for this step is that if the pixels of the OLED display and the SLM are perfectly aligned, then the dark region remains dark region and the bright region remains bright region.

The align result is shown in Figure S21.

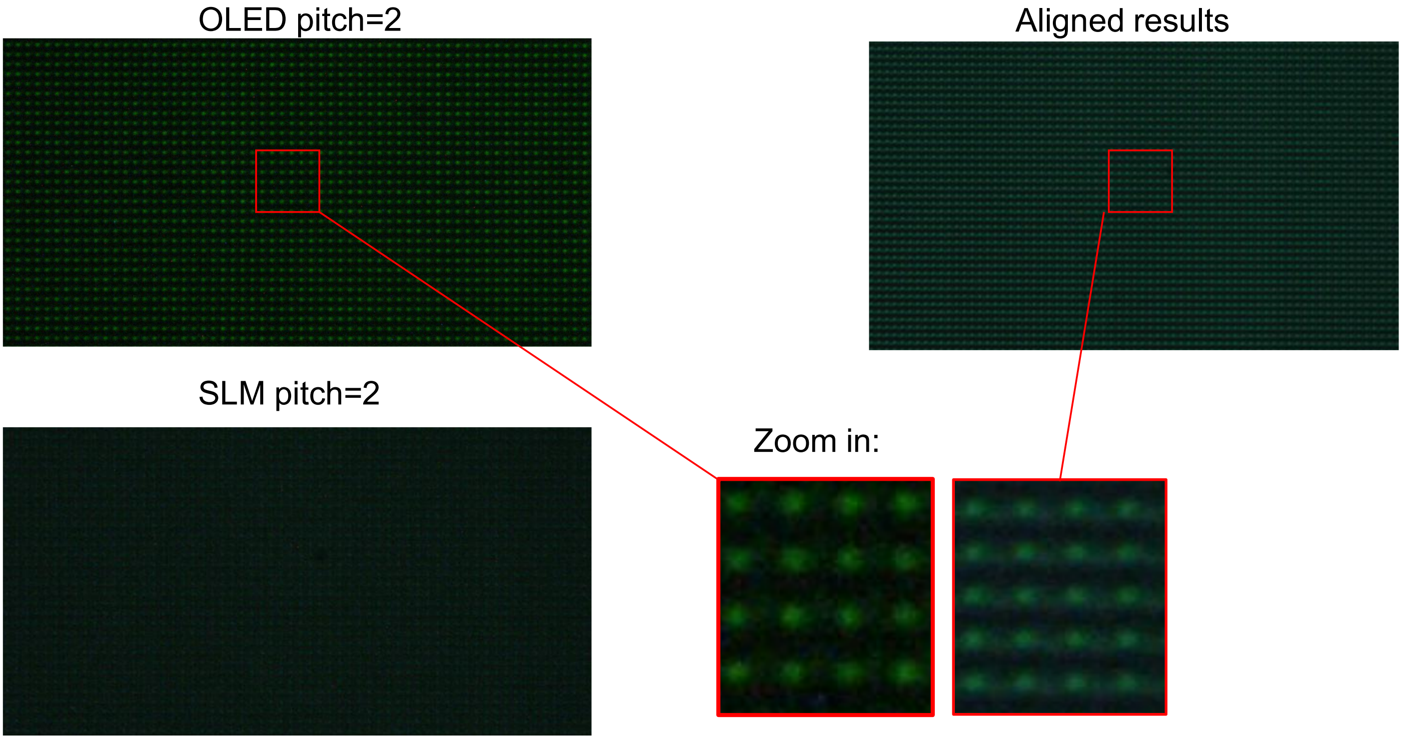

Figure S21. Alignment between OLED and SLM.

6.2. Details about training the neural network

We first select a two layer fully connected neural network to validate our method's effectiveness. The selected neural network consists of two fully connected layers (784×144, 144×10). The output of the first fully connected layer is activated by a sigmoid activation function. The fully connected layer's matrix multiplication operation is transformed into the way that imaging system operates without considering all kinds of noises. The learning rate is 0.0015 with a decay for every 10 training epochs. During the neural network training, we add a restrict function to restrict the weight value between -1 and 1. We first train the neural network with standard backpropagation (BP), the loss converges for 10 training epochs and the MNIST classification accuracy is 97.23% for 10,000 test pictures. However, the imaging system is very sensitive to misalignment issues. During the experiment, we use the built-in affine function within Pytorch to intentionally create misalignment between OLED and SLM. With the affine function, we can quantitatively modify the rotation angle, shift pixel number and scaling factor between OLED and SLM, and then measure the inference accuracy.

We first validate our method on increasing robustness against rotation. Since the training process does not consider rotation angle as a trainable parameter, thus we cannot get the gradient of loss with respect to rotation angle. However, we can measure the gradient through finite difference method by slightly perturbing the rotation angle. Here, we provide the pseudo code as shown below,

Pseudocode:

Input: Training set data $\mathbf{x}$.
Output: Model trained with SAT.
1. Initialize control weights $\mathbf{W}_0$, rotation angle $\theta_0, t = 0$;
2. While $not\ converged$ do
    Compute gradient of weights $\nabla_{\mathbf{w}} L(\mathbf{W}_{\mathrm{t}})$;
    Record the loss L_{t} with rotation angle θ_0;
    Perturb the rotation angle $\theta_0 + \delta\theta$;
    Record the loss L'_{t} with perturbation rotation angle.

Compute gradient of rotation angle $dL/d\theta = (L'_{\mathrm{t}} - L_{\mathrm{t}})/\delta\theta$;

Update rotation angle $\theta_{\mathrm{adv}} = \theta_0 + \alpha_1 \frac{dL/d\theta}{\|dL/d\theta\|_2}$

Update weights $\mathbf{W}_{\mathrm{adv}} = \mathbf{W}_{\mathrm{t}} + \alpha_2 \frac{\nabla_{\mathbf{W}} L(\mathbf{W}_{\mathrm{t}})}{\|\nabla_{\mathbf{W}} L(\mathbf{W}_{\mathrm{t}})\|_2}$;

Compute gradient $\nabla_{\mathbf{W}} L(\mathbf{W}_{\mathrm{adv}})$;

Update weights $\mathbf{W}_{\mathrm{t+1}} = \mathbf{W}_{\mathrm{t}} - \eta \nabla_{\mathbf{W}} L(\mathbf{W}_{\mathrm{adv}})$;

Recover the rotation angle to initial rotation angle θ_0;

t = t + 1;

End

Return $\mathbf{W}_t$

Note, during the simulation we restrict the θ_{adv} within $[-2.2°, 2.2°]$ to make sure the optimization converges.

The main difference between the new pseudo code and the previous one is that in the second back propagation stage, the rotation angle is recovered to the initial angle θ_0 rather than updated to the new value. The reason is that the rotation angle is not a trainable parameter in real imaging system, therefore we need to train the model that is robust against rotation angle when the angle is the initial angle. The optimization process goes the same way for increasing robustness against shift and scaling.

6.3. Joint optimization

Pseudocode:

Input: Training set data $\mathbf{x}$.

Output: Model trained with SAT.

1. Initialize control weights $\mathbf{W}_0$, rotation angle $\theta_0, t = 0$;
2. While $not\ converged$ do

    If t // 3 = 0

        Compute gradient of weights $\nabla_{\mathbf{W}} L(\mathbf{W}_\mathrm{t})$;

        Record the loss L_t with rotation angle θ_0;

        Perturb the rotation angle $\theta_0 + \delta\theta$;

        Record the loss L'_t with perturbation rotation angle;

        Compute gradient of rotation angle $dL/d\theta = (L'_\mathrm{t} - L_\mathrm{t})/\delta\theta$;

        Update rotation angle $\theta_\mathrm{adv} = \theta_0 + \alpha_1 \frac{dL/d\theta}{\|dL/d\theta\|_2}$

        Update weights $\mathbf{W}_\mathrm{adv} = \mathbf{W}_\mathrm{t} + \alpha_2 \frac{\nabla_{\mathbf{W}} L(\mathbf{W}_\mathrm{t})}{\|\nabla_{\mathbf{W}} L(\mathbf{W}_\mathrm{t})\|_2}$;

        Compute gradient $\nabla_{\mathbf{W}} L(\mathbf{W}_\mathrm{adv})$;

        Update weights $\mathbf{W}_\mathrm{t+1} = \mathbf{W}_\mathrm{t} - \eta \nabla_{\mathbf{W}} L(\mathbf{W}_\mathrm{adv})$;

        Recover the rotation angle to initial rotation angle θ_0;

    If t // 3 = 1

        Perturb the shift parameter;

    If t // 3 = 2

        Perturb the scaling parameter;

    t = t + 1;

End

Return $\mathbf{W}_\mathrm{t}$

Our experimental demonstration is single parameter optimization result. However, our method is also applicable to joint optimization of multiple parameters. We propose a sequential joint optimization strategy: optimizing the rotation angle in epoch 1, the pixel shift in epoch 2, and the scale factor in epoch 3. This approach enables joint consideration of all parameters while maintaining the same computational complexity as optimizing a single parameter at a time. To evaluate the trained model's robustness, first take rotation angle, shift pixel and scale factor as the joint optimization parameters. After training, we evaluate the model's robustness through a 2D sweep over rotation angle and shift pixel and keep the scale factor at 1. As shown in Figure S22, our method maintains high robustness against both rotation angle and shift pixel changes after the joint optimization. Next, the robustness of the model trained with joint parameter optimization is compared with that of models trained with single-parameter optimization for rotation angle, shift pixel, and scale factor, respectively. As shown in Figure S23, joint optimization achieves comparable robustness to that of single-parameter optimization. This result demonstrates that our method is applicable to joint optimization of multiple parameters without degrading the robustness of the trained model.

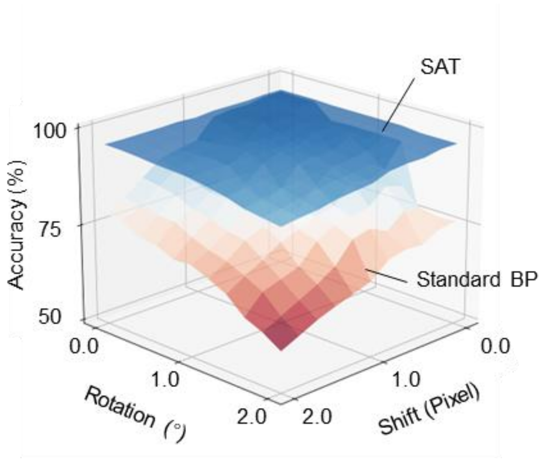

Figure S22. 2D accuracy sweep with different rotation angle and shift pixel.

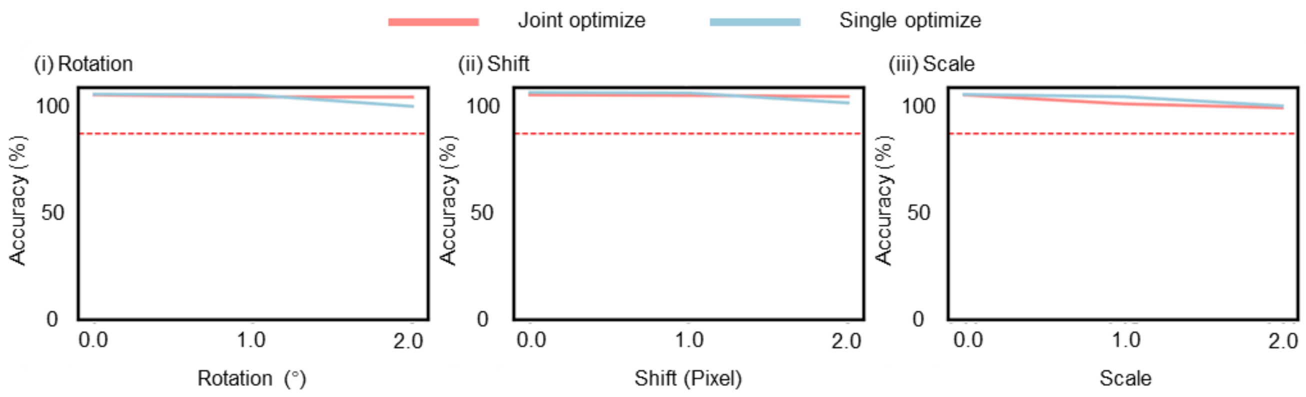

Figure S23. Inference accuracy with different (i) rotation angle, (ii) shift pixel and (iii) scaling number.

6.4. Full neural network inference capability simulation

The principle described in manuscript Section 2.1 demonstrates that SAT is universally applicable to systems with or without nonlinear functions. SAT identifies flat minima based on gradients, which can be approximated from an imperfect model or measured using finite-difference methods. Therefore, even for systems with nonlinearities, the gradient approximation process is the same as for systems with linear functions. Here, we include an additional simulation study on a deep diffractive neural network (D2NN). Here we follow the setting in [10] and include nonlinear functions between the linear diffractive neural network, as illustrated in Figure S24. The simulated system consists of two programmable linear layers and two nonlinear layers. The first nonlinear layer is implemented using a saturable absorber, which introduces an intensity-dependent transmission while preserving the optical phase [13]. And the second nonlinear component is the photodetector (PD) array, which introduces a quadratic nonlinearity during the optical-to-electrical conversion process. The input image is encoded via a spatial light modulator (SLM), after which light propagates through the layered structure (linear → nonlinear → linear → nonlinear) and is ultimately captured by the PD array. We apply this system to perform handwritten digits classification, where the output class is determined by the index of the maximum PD response.

We train the physical network using both standard backpropagation (BP) and our SAT. During training, we employ the finite difference method to approximate the gradient of the loss with respect to the rotation angle (which acts as trainable control parameters), and we intentionally introduce perturbations in rotation to evaluate robustness.

As shown in Figure S25, our method maintains high classification accuracy under rotation angle variations. These results clearly demonstrate that SAT remains effective when both linear and nonlinear operations are physically implemented. The detailed simulation parameters are depicted in Table.S8.

Table.S8 Simulation hyperparameters

Parameter name	Value
Batch size	200
Learning rate	0.002
Linear layers size	128*128
Linear layer pixel resolution	20 μm
Laser wavelength	532 nm

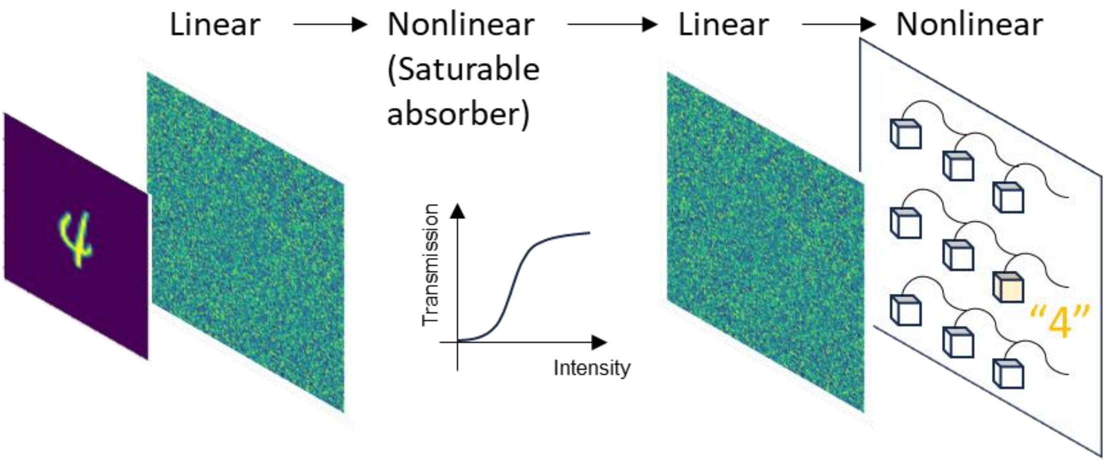

Figure S24. Schematic diagram of the simulated Deep diffraction neural network (D2NN).

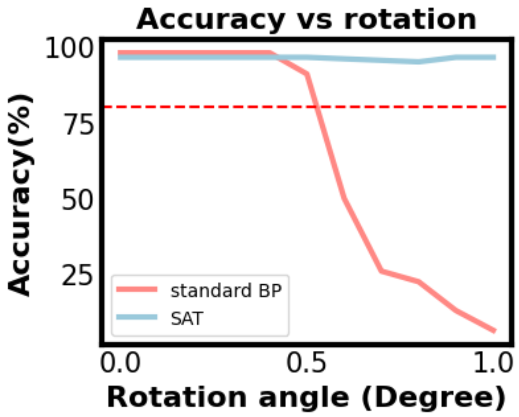

Figure S25. Inference accuracy with rotation angle change from 0.0° to 1.0°.

References

[1] Foret, P., Kleiner, A., Mobahi, H., & Neyshabur, B. (2020). Sharpness-aware minimization for efficiently improving generalization. arXiv preprint arXiv:2010.01412.

[2] Zhang, X., Xu, R., Yu, H., Zou, H., & Cui, P. (2023). Gradient norm aware minimization seeks first-order flatness and improves generalization. In Proceedings of the IEEE/CVF Conference on Computer Vision and Pattern Recognition (pp. 20247-20257).

[3] Yao, Z., Gholami, A., Keutzer, K., & Mahoney, M. W. (2020, December). Pyhessian: Neural networks through the lens of the hessian. In 2020 IEEE international conference on big data (Big data) (pp. 581-590). IEEE.

[4] Kariyappa, S., Tsai, H., Spoon, K., Ambrogio, S., Narayanan, P., Mackin, C., ... & Burr, G. W. (2021). Noise-resilient DNN: Tolerating noise in PCM-based AI accelerators via noise-aware training. IEEE Transactions on Electron Devices, 68(9), 4356-4362.

[5] Yang, X., Wu, C., Li, M., & Chen, Y. (2022). Tolerating noise effects in processing-in-memory systems for neural networks: a hardware–software codesign perspective. Advanced Intelligent Systems, 4(8), 2200029.

[6] Wu, C., Yang, X., Yu, H., Peng, R., Takeuchi, I., Chen, Y., & Li, M. (2022). Harnessing optoelectronic noises in a photonic generative network. Science advances, 8(3), eabm2956.

[7] Mourgias-Alexandris, G., Moralis-Pegios, M., Tsakyridis, A., Simos, S., Dabos, G., Totovic, A., ... & Pleros, N. (2022). Noise-resilient and high-speed deep learning with coherent silicon photonics. Nature communications, 13(1), 5572.

[8] Xu, T., Zhang, W., Zhang, J., Luo, Z., Xiao, Q., Wang, B., ... & Huang, C. (2024). Control-free and efficient integrated photonic neural networks via hardware-aware training and pruning. Optica, 11(8), 1039-1049.

[9] Wright, L. G., Onodera, T., Stein, M. M., Wang, T., Schachter, D. T., Hu, Z., & McMahon, P. L. (2022). Deep physical neural networks trained with backpropagation. Nature, 601(7894), 549-555.

[10] Zheng, Z., Duan, Z., Chen, H., Yang, R., Gao, S., Zhang, H., ... & Lin, X. (2023). Dual adaptive training of photonic neural networks. Nature Machine Intelligence, 5(10), 1119-1129.

[11] Clements, W. R., Humphreys, P. C., Metcalf, B. J., Kolthammer, W. S., & Walmsley, I. A. (2016). Optimal design for universal multiport interferometers. Optica, 3(12), 1460-1465.
orks trained with backpropagation. Nature, 601(7894), 549-555.
[12] Wang, T., Ma, S. Y., Wright, L. G., Onodera, T., Richard, B. C., & McMahon, P. L. (2022). An optical neural network using less than 1 photon per multiplication. Nature Communications, 13(1), 123.

[13] Lin, X., Rivenson, Y., Yardimci, N. T., Veli, M., Luo, Y., Jarrahi, M., & Ozcan, A. (2018). All-optical machine learning using diffractive deep neural networks. Science, 361(6406), 1004-1008.
